# Evaluation of Fluorescence-Based Screening Assays for the Detection and Quantification of Silyl Hydrolase Activity

**DOI:** 10.1021/acsomega.4c05409

**Published:** 2024-06-25

**Authors:** Jason Z. He, Yuqing Lu, Neha Jain, David G. Churchill, Lu Shin Wong

**Affiliations:** † Manchester Institute of Biotechnology, The University of Manchester, 131 Princess Street, Manchester M1 7DN, U.K.; ‡ Department of Chemistry, The University of Manchester, Oxford Road, Manchester M13 9PL, U.K.; § Department of Chemistry, Korea Advanced Institute of Science and Technology, Daejeon 34141, Republic of Korea

## Abstract

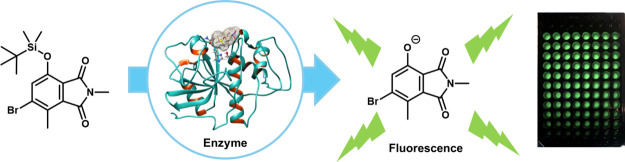

This study reports the development of fluorometric assays for the detection and quantification of silyl hydrolase activity using silicatein as a model enzyme. These assays employed a series of organosilane substrates containing either mycophenolate or umbelliferone moieties, which become fluorescent upon hydrolysis of a scissile Si–O bond. Among these substrates, the mycophenolate-derived molecule MycoF, emerged as the most promising candidate due to its relative stability in aqueous media, which resulted in good differentiation between the enzyme-catalyzed and uncatalyzed background hydrolysis. The utility of MycoF was also demonstrated in the detection of enzyme activity in cell lysates and was found to be capable of qualitative identification of positive “hit” candidates in a high-throughput format. These fluorogenic substrates were also suitable for use in quantitative kinetic assays, as demonstrated by the acquisition of their Michaelis–Menten parameters.

## 1 Introduction

Organosilanes represent a major class of compounds with diverse applications in materials chemistry as silicone polymers^1−3^ and as auxiliaries in the multistep chemical synthesis of complex molecules.^4,5^ In particular, silyl groups are widely used as protecting groups for hydroxy moieties due to their inertness toward a range of conditions.^6,7^ However, the introduction of organosilyl groups is often dependent on silane precursors (e.g., chlorosilanes, hydrosilanes), the production of which is energy-intensive and generates waste streams that are environmentally undesirable.^8,9^

Efforts to improve the sustainability of organic chemistry have involved the harnessing of enzymes for synthetic transformations in which reactions proceed under benign conditions and generally with high selectivity.^10^ Previous investigations have demonstrated that a range of hydrolases such as proteases, esterases, and lipases are capable of catalyzing the hydrolysis and condensation of Si–O bonds.^11,12^ In recent years, silicatein enzymes derived from marine demosponges have also garnered particular interest. These silicateins are one of the few enzymes that are involved in silicon metabolism, and catalyze the condensation of dissolved silicates into silica, which is incorporated into the inorganic skeleton of these organisms.^13^ The most abundant isoform of this family, silicatein-α (Silα), has also been shown to catalyze the hydrolysis and condensation of various organic silyl ethers, including those that were not accepted by the previously studied hydrolases.^14^ Therefore, these enzymes may be good candidates for the development of biocatalysts for the synthetic manipulation of organosiloxanes, including silicone polymers.^15^

To recombinantly engineer existing enzymes or enable the discovery of new enzymes for these reactions, methods to detect and quantify the biocatalytic hydrolysis of Si–O bonds are required. In these biocatalyst development efforts, the screening of large libraries of enzymes (ca. 10^4^–10^7^ individual candidates) is typically executed using cell lysate extracts^16^ that require a high-throughput screening method, such as absorbance or fluorescence spectroscopy.

Silyl ether substrates encompassing the 4-nitrophenol derivatives have been developed for continuous assays by UV–vis spectrophotometry,^17,18^ whereby hydrolysis of the Si–O bond results in the release of highly absorbing nitrophenolate ions. However, these anions absorb in the ∼400 nm region (i.e., in the yellow region of the visible spectrum), which also overlaps with the typical absorption spectra and color of crude cell supernatants,^19^ so these reagents are therefore unsuitable for screening experiments involving such cell lysates. An alternative strategy is required in which only the target hydrolysis product is detected, with minimal interference from other cellular constituents.

Herein is reported the development of a fluorometric assay for the hydrolysis of Si–O bonds. Subsequently, their application in the context of analyzing crude enzyme extracts was investigated. Finally, a kinetic analysis of a Silα fusion protein was conducted to demonstrate the application of these reagents in the determination of Michaelis–Menten enzyme kinetics parameters.

## 2 Results and Discussion

### 2.1 Design and Rationale of Fluorogenic Substrates

**Scheme 1:**
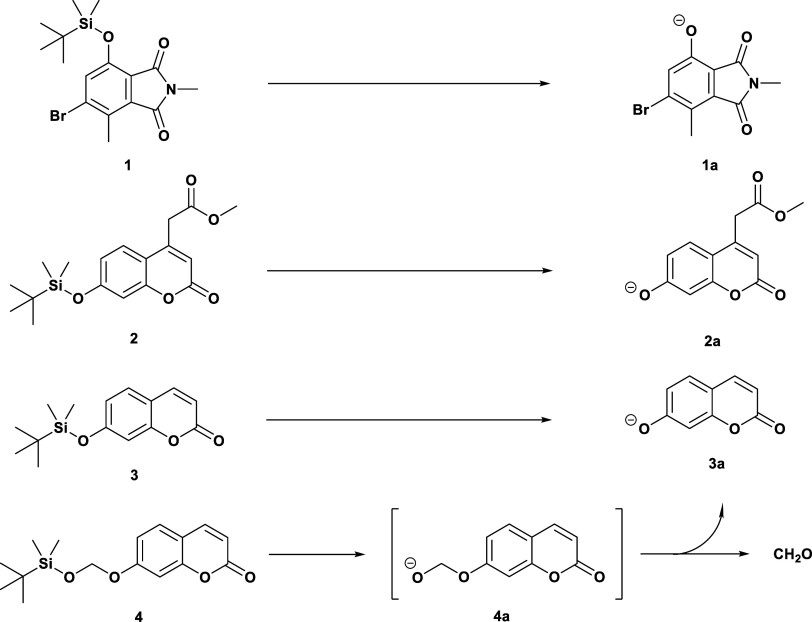
Molecular Structures and Hydrolysis Reactions for the Silyl Ether Enzyme Substrates **1***–***4** Presented in This Work

Conceptually, fluorometric enzyme assays involve the use of a nonfluorescent reagent molecule as the enzyme substrate that is designed such that the occurrence of the chemical transformation of interest results in the formation of a fluorescent product, which is then detected and quantified spectrophotometrically. In the context of this study, the enzyme substrates all incorporated a silyl ether, which upon cleavage of the Si–O bond releases the fluorophore (Scheme 1). To explore suitable candidate substrates, the fluorogenic compounds **1** and **2**, which have been reported as chemosensors for the detection of fluoride ions within living cells, were first selected.^20,21^ The mycophenolic acid-derived “MycoF” **1** was of particular interest in this context due to its small size, relative stability under physiological conditions, and a large Stokes’ shift that minimizes the re-absorption of emitted photons. In both molecules, the silyl component consisted of a *tert*-butyldimethylsilyl (TBDMS) group, consistent with the previously described colorimetric substrates.^17^

A further two silylated umbelliferone derivatives, **3** and **4**, inspired by the fluorogenic substrates for esterase detection,^22,23^ were also synthesized and tested. The silyl ethers of nitrophenols and umbelliferones are susceptible to a significant degree of background hydrolysis, even in the absence of the biocatalyst. This aqueous instability can be attributed to the nucleofugality of the phenolate leaving group and, hence, the lability of the Si–O bond. In an effort to address this issue, the incorporation of an oxymethyl moiety in substrate **4** aimed to enhance the overall stability of the compound such that cleavage of the Si–O bond first yields the hemiacetoxy species **4a**, a poorer leaving group. Compound **4a** then spontaneously collapses to produce umbelliferone (**3a**) and formaldehyde byproduct (Scheme 1).

### 2.2 Substrate Screening

To investigate the utility of these substrates, enzyme assays were carried out using Silα, fused with trigger factor at the N-terminal and a Strep-tag II affinity tag at the C-terminal (henceforth referred to as TF-Silα-Strep), as the model enzyme. Initially, the assays were carried out under the same conditions as the previously reported 4-nitrophenoxy substrates.^17,18^ Negative control reactions were also carried out where the enzyme was omitted, to evaluate the stability of each substrate toward aqueous (nonenzymatic) background hydrolysis.

**Figure 1:**
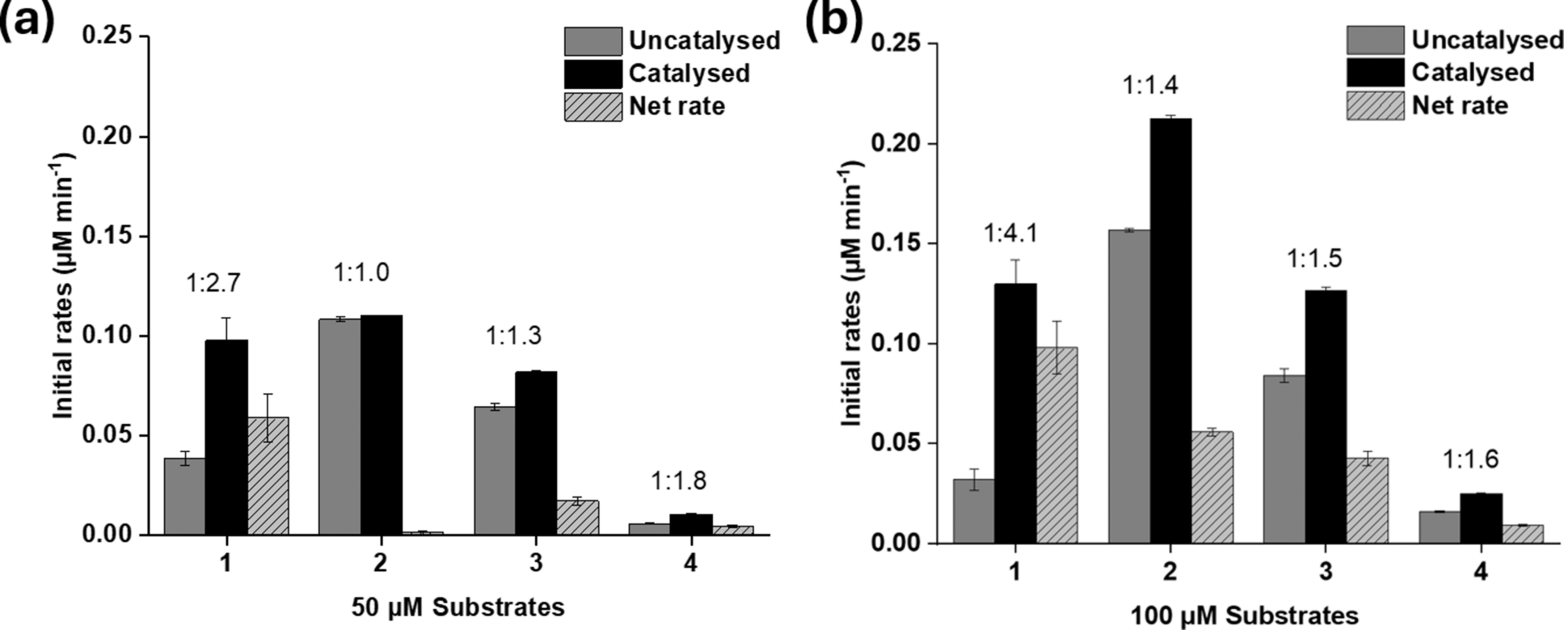
Bar charts representing the initial rates of enzyme-catalyzed, uncatalyzed (enzyme omitted), and net hydrolysis for **1***–***4**, at (a) 50 or (b) 100 μM substrate concentrations. The ratios of the catalyzed to uncatalyzed reactions are given above each column group. Net rates were calculated as the difference between the uncatalyzed and catalyzed rates. Enzymatic reactions were carried out with 6.7 μM TF-Silα-Strep, 10% v/v 1,4-dioxane, 50 mM Tris buffer at pH 8.5, and 100 mM NaCl. Error bars represent the ±1 SEM from triplicate experiments.

The initial rates of hydrolysis were obtained (Figures 1, S1, S2, and Table S1 in the SI) and it was revealed that all the substrates were susceptible to a certain degree of uncatalyzed hydrolysis. In general, **4** gave the lowest absolute rates of reaction for both catalyzed and uncatalyzed reactions, consistent with the intended design. Conversely, substrate **2** gave the highest absolute rates. Substrate **1** gave the best differentiation between the enzymatic and uncatalyzed reactions, with a relatively high catalyzed rate of hydrolysis (similar to that of **2**) but a comparatively lower uncatalyzed rate. In quantitative terms, this substrate gave the largest ratio of catalyzed to uncatalyzed rates and the largest net rate of reaction; with a ratio of 4.1 and net rate of 0.0980 μM min^–1^ being achieved in assays using 100 μM substrate. In contrast, **2** and **3** showed relatively small differences in the rates of enzymatic and background hydrolysis (ratios of <1.5 in both cases), suggesting these molecules may not be accepted as substrates by TF-Silα-Strep. A possible reason could be due to the relatively large size of the umbelliferone moiety, which, on the inclusion of the additional acetoxy methyl ester group, led to an even greater bulk of the fluorophore. On comparing **3** and **4**, the net rate was approximately four-fold greater for the former regardless of substrate concentration, confirming the higher stability conferred by the incorporation of the oxymethyl moiety in **4**, such that even the enzyme was ineffective at catalyzing the hydrolysis.

On comparing the effect of increasing substrate concentration from 50 to 100 μM, a moderate improvement in the rate ratios was observed with all the substrates. It was found that **1** gave the largest increase in the ratio compared with the other substrates. The net hydrolytic rate of **1** increased to a lesser extent when the substrate concentration was increased, while it was the greatest for **2**. These observations appear to reflect the differences in the binding strength of the substrate to the enzyme active site and the concentration necessary to attain enzyme saturation (see below).

### 2.3 pH Optimization of Assay with **1**

**Figure 2:**
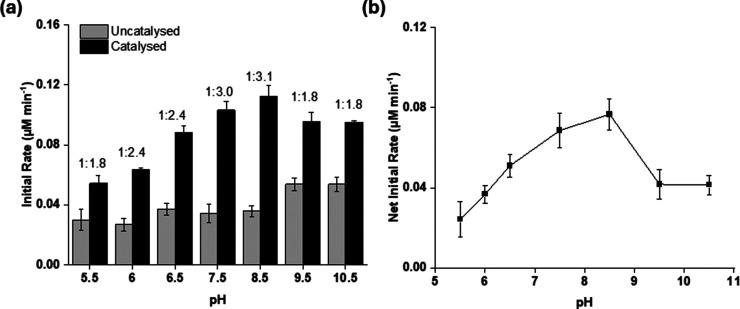
Bar chart of initial rate of hydrolysis of **1** against variation of pH for (a) uncatalyzed reaction (absence of enzyme) and catalyzed (presence of enzyme) and (b) plot of the net enzymatic rate of hydrolysis. The net rates were calculated by the difference between the catalyzed reaction rate and the uncatalyzed reaction rate. The ratios of the catalyzed to uncatalyzed reaction are provided above each column pair. Enzymatic reactions were carried out with 6.7 μM TF-Silα-Strep, 50 μM substrate **1**, 10% v/v 1,4-dioxane, 50 mM sodium acetate (for pH 5.5) or Tris (all other pH values) buffer, and 100 mM NaCl. The error bars represent ±1 SEM from triplicate experiments.

As compound **1** gave the best differentiation between the enzymatic and nonenzymatic reactions, the effect of pH on assays involving this substrate was further investigated, since this experimental parameter can have a significant effect on the fluorescence intensity and enzyme activity. Here, it was found that the best enzyme activity (as evidenced by the highest net reaction rate) occurred at pH 8.5 (Figure 2), which was in agreement with the previous studies.^18^ In general, the nonenzymatic rate of hydrolysis was broadly similar across the pH range with only a slight increase toward the higher pH levels. This result conforms with the previous study of this compound where the increase in basicity resulted in greater levels of fluorescence.^21^ This pH profile of TF-Silα-Strep also aligns with other “alkaline” serine proteases such as trypsin and chymotrypsin^24,25^ that have their highest activity at pH levels >7.

### 2.4 Fluorometric Assays in *E. Coli* Cell Lysate

**Figure 3:**
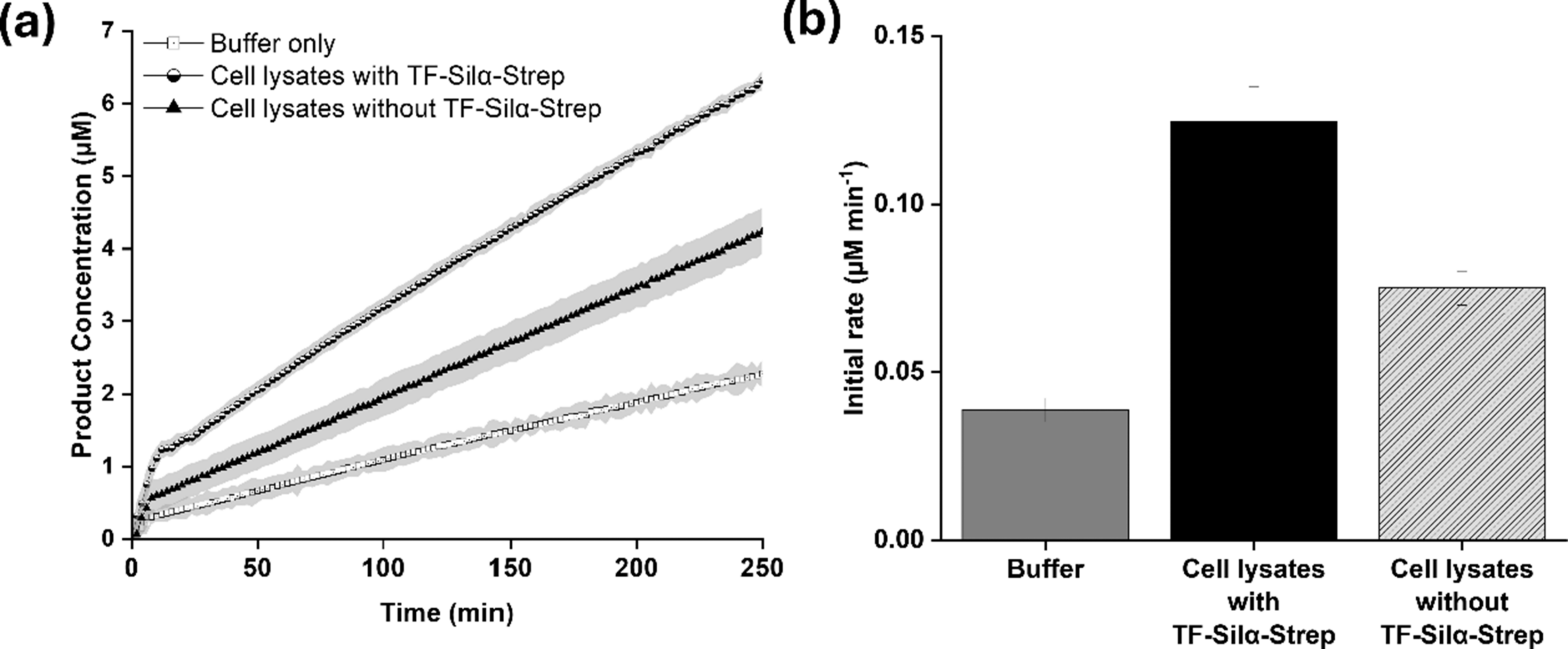
(a) Graph of average product concentration against reaction time for the hydrolysis of **1** in cell lysates. (b) Bar chart of initial hydrolysis rates in the reaction buffer, reactions containing cell lysates expressing TF-Silα-Strep and cell lysates not expressing the enzyme. Assays were carried out in Tris buffer (50 mM Tris, 100 mM NaCl at pH 8.5), substrate **1** (100 μM), 10% v/v 1,4-dioxane and 0.5 mg mL^–1^ of lysate protein. The shaded area in (a) represents ±1 SEM of each time point. The error bars in (b) represent ±1 SEM from triplicate experiments.

Next, the applicability of compound **1** for the selective detection of silyl hydrolase activity in cell lysates was investigated. Here, clarified lysate from *E. coli* cells expressing the gene for TF-Silα-Strep were used as the source of the enzyme and compared to lysates that did not express this gene product (i.e., cells did not contain the plasmid carrying this gene). It was found that the hydrolysis of **1** in the presence of lysates containing TF-Silα-Strep indeed resulted in a higher rate of product generation (Figure 3). However, for lysates without the desired enzyme, the rate of hydrolysis was still two-fold greater than that for aqueous hydrolysis (i.e., buffer only). This indicates that even in the absence of the target enzyme, other constituents in the lysate were able to catalyze general (nonspecific) acid–base hydrolysis.

Nevertheless, an examination of the time course of product formation (Figure 3a) showed that cell lysates with TF-Silα-Strep afforded a fluorescence intensity (and hence product concentration) that was approximately two-fold greater than that of the lysate without the enzyme. To determine whether this difference (and taking into account the statistical effect size) was sufficient to allow this assay to be used in a high-throughput screen, the *Z*-factor^26^ at each time point was then calculated. Here, the *Z*-factor was used to evaluate the separation of signals between the positive and negative samples, which in this case were the cell lysates containing enzymes and without enzymes, respectively. It was found that a *Z*-factor of >0 (i.e., sufficient for a qualitative assay) was achieved after 90 min (Table S2 in the SI). It should be noted that the TF-Silα-Strep that was used as the model enzyme here has a relatively low activity (see Michaelis–Menten kinetics data below) and was deliberately chosen to demonstrate the detection of even low levels of biocatalysis in a complex lysate mixture. It is anticipated that with more active enzymes, *Z*-factor values of >0 would be achieved within substantially shorter timeframes, or to allow higher *Z*-factors (i.e., greater confidence in the identification of hits) with the same amount of time.

### 2.5 Kinetic Evaluation of Silyl Ether Hydrolysis by TF-Silα-Strep

To further characterize the enzyme-catalyzed hydrolysis of these substrates and demonstrate their applicability for the quantification of enzyme kinetics, a series of assays were conducted to extract their Michaelis–Menten parameters with respect to TF-Silα-Strep (Table 1). It was found that the *K*_M_ values were in the micromolar range, which was similar to that for the previously described nitrophenoxy silyl compounds with the same enzyme.^17,27^ The general trend with the current substrates appeared to correlate with increasing steric bulk, with umbelliferone methyl ester **2** and mycophenolate **1** displaying the highest and lowest *K*_M_, respectively. These values support the results of the initial screening above, suggesting that **2** was not a good substrate for this particular silicatein enzyme.

**Table 1:** Table of Michaelis–Menten Parameters for TF-Silα-Strep with Respect to Compounds **1**–**4**^a^

substrate	*K*_M_ (μM)	*k*_cat_ (min^–1^)	*k*_cat_/*K*_M_ (10^–4^ min^–1^ μM^–1^)
**1**	12.46 ± 1.81	0.0142 ± 0.0002	11.24
**2**	187.06 ± 40.47	0.0245 ± 0.0035	1.35
**3**	77.76 ± 13.06	0.0223 ± 0.0015	2.95
**4**	63.95 ± 25.59	0.0035 ± 0.0006	0.55

a The data and plots from which these values are derived are given in Figures S3 and S4 in the SI. Assays were performed in 10% v/v 1,4-dioxane, 50 mM Tris, and 100 mM NaCl, pH 8.5.

For the catalytic turnover (*k*_cat_), it was observed that the greatest rate was achieved by substrate **2**. However, the higher *K*_M_ for this substrate meant that the overall catalytic efficiency (*k*_cat_/*K*_M_) was inferior to those of the other substrates. In contrast, for compound **1**, a moderate *k*_cat_ combined with the best *K*_M_ afforded the best overall efficiency. Oxymethyl-containing **4** afforded the lowest overall efficiency due to the very low value of *k*_cat_. This result is consistent with previous studies that investigated the condensation of silanols and alcohols to form silyl ethers (i.e., the reverse reaction to the hydrolysis), which showed that aliphatic alcohols are not accepted by TF-Silα-Strep.^14^

## 3 Conclusions

In summary, this study reports the design and evaluation of fluorogenic organosilyl ether molecules for the screening and quantification of silyl hydrolase activity. Among the substrates that were investigated, MycoF (**1**) emerged as the most promising candidate, exhibiting the highest ratio between the enzymatic hydrolytic and the background hydrolytic rates due to its relative stability in aqueous conditions. This reagent molecule can be employed in a qualitative screening format for crude cell lysates, though care must be taken to define the criteria for identifying positive hits. Additionally, these fluorogenic molecules can also be used for determining the Michaelis–Menten enzyme kinetics parameters of silyl hydrolases. Although **1** was found to be the most effective substrate for the TF-Silα-Strep model enzyme used in this study, the variations in the kinetic parameters show that substrates should be selected to match the enzyme of interest.

It is envisaged that these assays may also contribute to furthering research in many areas. Apart from sponges, many marine organisms,^28,29^ terrestrial plants,^30^ and microbes^31^ are also known to use and manipulate silicon, and their sequestration of silicon represents a major reservoir of this element in the Earth’s biogeochemical cycle.^29,32,33^ Thus, apart from the engineering of new biocatalysts for sustainable silicon chemistry and the characterization of new hydrolase enzymes, these assays may also be applicable in studies aimed at understanding the metabolism of silicon in living organisms.

## 4 Methods and Materials

### 4.1 Materials and Equipment

All solvents and reagents were of analytical grade and purchased from Sigma-Aldrich, Fluorochem, VWR, TCI, or Fisher Scientific. All buffer solutions were prepared with deionized water. The Coomassie dye solution for the Bradford assays was obtained from Sigma-Aldrich. The substrate **1** and its corresponding hydrolysis product **1a** were synthesized as previously reported.^21^ Substrates **2**–**4** were synthesized according to the procedures described below (the synthetic routes for **2** and **4** are shown in Schemas S1 and S2 in the SI).

Cell lysis was performed with a Sonopuls HD 3100 ultrasonically homogenizer (Bandelin Electronic, Berlin, Germany). Streptavidin affinity chromatography was conducted using StrepTrap HP columns on an AKTA purification system (GE Healthcare, Amersham, UK). Fluorimetry was conducted using Synergy H1 and HT microtiter plate readers (BioTek Instruments, Winooski, VT, USA) and reactions were conducted in 96-well black microtiter plates (Greiner Bio-One, Stonehouse, UK). Buffer exchange was performed with PD-10 columns (Cytiva, Marlborough, MA, USA).

### 4.2 Production and Isolation of TF-Silα-Strep

For the production of the TF-Silα-Strep enzyme, *E. coli* BL21(DE3) cells transformed with pET11a-tf-sila-strep were grown at 37 °C overnight in 10 mL of lysogeny broth (LB) with added ampicillin (0.1 mg mL^–1^). The resulting culture was used to inoculate 500 mL LB, which was incubated at 37 °C to an optical density (OD600) of 0.6. One mM Isopropyl β-D-1-thiogalactopyranoside solution (500 μL) was added to induce the protein expression and the culture was shaken overnight at 20 °C (180 rpm). The cells were sedimented by centrifugation (4000 rpm for 30 min at 4 °C), the media was decanted, and the cell pellet was resuspended with Tris buffer (50 mM Tris, 100 mM NaCl, pH 8.5). The cells were lysed by ultrasonication, the lysate was filtered through a 0.22 μm pore syringe filter, and the protein was isolated by loading the clarified lysate onto the FLPC with a 5 mL StrepTrap column that had already been previously equilibrated with the binding buffer (50 mM Tris, 100 mM NaCl, pH 8.5). The protein was eluted with the elution buffer (50 mM Tris, 100 mM NaCl, and 50 mM Biotin, pH 8.5). Isolation of the desired protein was confirmed by SDS-PAGE (Figure S5 in the SI), and the fractions containing the protein were pooled and dialyzed overnight in the binding buffer at the desired pH (for pH 6.0–10.5). For pH 5.5, the eluted enzyme solution was exchanged with a buffer of 50 mM sodium acetate and 100 mM NaCl at this pH with PD-10 columns. The concentration of the isolated protein was estimated by UV*–*vis absorbance at 280 nm using the molar absorption coefficient (ε = 67700 M^*–*1^ cm^*–*1^) and the molecular weight (MW = 74,600 kDa) of the protein. If required, the enzyme solutions were diluted to the required concentration (13.4 μM) for subsequent assays by the addition of the relevant assay buffer (see below).

### 4.3 Fluorometric Assays with Enzymes

The fluorometric assay design was based on methods previously reported for the spectrophotometric assays.^17,18,27^ First, stock solutions (5 mM) of each fluorogenic substrate (**1**-**4**) were prepared in 1,4*-*dioxane and these were diluted with the same solvent to the concentrations needed for each assay. Into each well of a 96*-*well plate were added 80 μL of the assay buffer (50 mM Tris, 100 mM NaCl for pH 6.0–10.5; or 50 mM sodium acetate, 100 mM NaCl for pH 5.5) and 100 μL of TF-Silα-Strep enzyme (13.4 μM) solution. Twenty microliter aliquots of the substrate solutions of the appropriate concentration were added to give the final desired concentration of substrates and 6.7 μM of enzyme in a final assay volume of 200 μL. For the nonenzymatic reactions, the enzyme solution was omitted and 180 μL of the assay buffer was added to each well instead. The increase in fluorescence was measured using excitation and emissions wavelengths: 398 and 518 nm for **1**; 360 and 465 nm for **2**; and 360 and 460 nm for **3** and **4**; at 2 min intervals with the plate shaken at room temperature (typically 22–25 °C) between each data collection.

The corresponding fluorogenic products **1a***–***3a** were quantified with calibration curves (Figures S6–S8 in the SI) constructed from known concentrations of the product compounds dissolved in the relevant assay buffer. The initial rate (*V*_0_) was obtained by the steepest linear region of the curve as close as possible to the origin for 5–10 data points (10*–*20 min) with all experiments being carried out in technical triplicates.

For Michaelis–Menten calculations, the initial rates of a series of reactions across a range of substrate concentrations (typically between 10–500 μM) were obtained. These rates were plotted against substrate concentration and fitted to the Michaelis–Menten equation in OriginPro 2024 data analysis and graphing software (OriginLab, Northampton, MA, USA) to obtain the *K*_M_ and *V*_max_ values. The *k*_cat_ values were then calculated using the formula *k*_cat_ = *V*_max_/[*E*].

### 4.4 Cell Lysate Assays

Cell lysates containing TF-Silα-Strep were prepared according to the procedures described above. For cell lysates without TF-Silα-Strep, BL21(DE3) cells bearing no plasmid with the gene were initially cultured and shaken overnight at 37 °C in LB medium (10 mL) in the same manner but without added ampicillin. The cells were used to inoculate 500 mL of LB medium and grown at 37 °C overnight. The cells were sedimented, lysed, and centrifuged in the same manner as the cells bearing the enzymes to prepare the clarified cell lysate. The protein concentration within these lysates was determined using the Bradford assay^34^ and calibrated with known amounts of bovine serum albumin (BSA). For the fluorometric assays, the protein samples were diluted where necessary using the same assay buffer (50 mM Tris and 100 mM NaCl, pH 8.5) to a total protein concentration of 1.0 mg mL^–1^. Assays were performed in a similar manner as above, with 80 μL of assay buffer, 100 μL of cell lysate solution, and 20 μL of **1** (1 mM dissolved in 1,4-dioxane).

### 4.5 Synthesis of Substrate Candidates

#### 4.5.1 Methyl-2-(7-hydroxy-2-oxo-2*H*-chromen-4-yl)acetate, **2a**

To a solution of 2-(7-hydroxy-2-oxo-2*H*-chromen-4-yl)acetic acid (1607 mg, 6.8 mmol) in methanol (40 mL) was added thionyl chloride (1.0 mL). The reaction mixture was stirred at room temperature for 16 h, and the white precipitate was filtered off and washed with ethyl acetate to afford the methyl ester **2a** as white crystals. Yield: 1186 mg, 74%; δ_H_ NMR (500 MHz, DMSO) 10.67 (s, 1H), 7.58 (d, *J* = 8.7 Hz, 1H), 6.86 (dd, *J* = 8.7, 2.4 Hz, 1H), 6.79 (d, *J* = 2.4 Hz, 1H), 6.30 (s, 1H), 4.01 (s, 2H), 3.71 (s, 3H); δ_C_ NMR (126 MHz, DMSO) 170.14 (C=O), 161.76 (C=O), 160.60 (Ar C–O), 155.51 (Ar C–O), 149.99 (C=C), 127.20 (Ar C–H), 113.53 (Ar C–H), 112.64 (C=C), 111.67 (Ar C–H), 102.81 (Ar C–H), 52.67 (C–O), 37.10 (C–C); *m*/*z* (APCI^–^) 233 (100%, [M–H]^−^). The data are consistent with literature values.^35^

#### 4.5.2 Methyl-2-(7-((*tert*-butyldimethylsilyl)oxy)-2-oxo-2*H*-chromen-4-yl)acetate, **2**

To a solution of compound **2a** (500 mg, 2 mmol) in anhydrous DMF (10 mL) was added imidazole (270 mg, 4 mmol) and *tert*-butyldimethylchloride (603 mg, 4 mmol). The reaction was stirred at room temperature for 16 h and was found to have reached completion after this time by TLC. The reaction mixture was diluted with ethyl acetate (20 mL) and washed with water (10 mL) and then brine (10 mL). The combined organic extracts were dried in MgSO_4_ and evaporated under reduced pressure. The residue was purified by silica gel chromatography (hexane/ethyl acetate, 3:1) to afford the product as a yellow oil. Yield: 431 mg, 56%; *R*_f_ 0.45 (hexane/ethyl acetate, 3:1); δ_H_ NMR (400 MHz, CDCl_3_) 7.46 (d, *J* = 8.4 Hz, 1H), 6.81 (d, *J* = 8.3 Hz, 2H), 6.25 (s, 1H), 3.75 (s, 2H), 1.01 (s, 9H), 0.27 (s, 6H); δ_C_ NMR (101 MHz, CDCl_3_) 169.24 (C=O), 160.84 (C=O), 159.50 (Ar C–O), 155.26 (Ar C–O), 147.90 (C=C), 125.45 (Ar C–H), 117.45 (Ar C–H), 114.13 (Ar C–C), 113.07 (C=C), 107.98 (Ar C–H), 52.73 (C–O), 38.03 (C–C), 25.56 (Si–C–CH_3_), 18.27 (Si–C–CH_3_), −4.38 (Si–CH_3_); *m*/*z* (APCI^+^) 349 (100%, [M + H]^+^). The data are consistent with literature values.^20^

#### 4.5.3 7-((*tert*-Butyldimethylsilyl)oxy)-2*H*-chromen-2-one, **3**

A stirred solution of 7-hydroxy-2*H*-chromen-2-one (994 mg, 6.12 mmol) in anhydrous DMF (10 mL) was cooled to 0 °C under a nitrogen atmosphere, and imidazole (12.24 mmol, 840 mg) was added. The resulting solution was stirred for 20 min, and *tert*-butyldimethylsilyl chloride (1836 mg, 12.24 mmol) in DMF (10 mL) was added dropwise over a period of 15 min. The reaction mixture was warmed to 23 °C and stirred until TLC analysis indicated completion (approximately 16 h). The crude reaction was poured into water (50 mL) and extracted with ethyl acetate (3 × 40 mL). The combined organic layers were dried in Na_2_SO_4_ and concentrated under reduced pressure. The crude product was purified by silica gel chromatography to afford the product as a white solid. Yield: 696 mg, 70%; *R*_f_ 0.52 (hexane/ethyl acetate, 10:1); δ_H_ (400 MHz; CDCl_3_) 7.63 (d, *J* = 9.5 Hz, 1H), 7.33 (d, *J* = 8.2 Hz, 1H), 6.81–6.73 (m, 2H), 6.25 (d, *J* = 9.5 Hz, 1H), 0.99 (s, 9H), 0.25 (s, 6H). δ_C_ (101 MHz; CDCl_3_) 161.19 (C=O), 159.39 (Ar C–H), 155.60 (Ar C–H), 143.38 (C=C), 128.72 (Ar C–H), 117.47 (Ar C–H), 113.40 (Ar C–H), 113.2 (C=C), 107.76 (Ar C–H), 25.57 (Si–C–CH_3_), 18.28 (Si–C–CH_3_), −4.39 (Si–CH_3_); *m*/*z* (ESI^+^) 277 (100%, [M + H]^+^). The data are consistent with literature values.^36^

#### 4.5.4 7-((Methylthio)methoxy)-2*H*-chromen-2-one, **4b**

A dry flask containing a solution of 7-hydroxy-2*H*-chromen-2-one (800 mg, 5 mmol) in anhydrous DMF (10 mL) was cooled in an ice bath. NaH (240 mg, 10 mmol) was then slowly added, followed by chlorodimethylsulfide (630 mg, 6.5 mmol) after 10 min. After being stirred for 4 h, the reaction mixture was poured into crushed ice and extracted with ethyl acetate (3 × 10 mL). The combined organic extracts were dried over MgSO_4_ and evaporated under reduced pressure. The residue was purified by silica gel chromatography (hexane/ethyl acetate, 3:2) to afford the product as yellow crystals. Yield: 950 mg, 85.5%; *R*_f_ 0.59 (hexane/ethyl acetate, 3:2); ν_max_(soild)/cm^–1^ 2921 (C–H), 1750 (C=O), 1251 (C–O); δ_H_ (400 MHz; CDCl_3_) 7.64 (d, *J* = 9.4 Hz, 1H), 7.40 (d, *J* = 9.3 Hz, 1H), 6.92–6.85 (m, 2H), 6.28 (d, *J* = 9.4 Hz, 1H), 5.20 (s, 2H), 2.27 (s, 3H)·; δ_C_ (101 MHz; CDCl_3_) 160.81 (C=O), 159.95 (Ar C–O), 155.38 (Ar C–O), 143.07 (C=C), 128.60 (Ar C–H), 113.54 (Ar C–H), 113.32 (Ar C–H), 113.09 (C=C), 103.01 (Ar C–H), 72.52 (S–C–O), 14.53 (S–CH_3_); *m*/*z* (APCI^+^) 223 ([M + H]^+^, 100%); HRMS calculated for C_11_H_10_O_3_S [M + H]^+^ 223.0423, found 223.0423, δ <0.2 ppm.

#### 4.5.5 7-(Chloromethoxy)-2*H*-chromen-2-one, **4c**

A dry flask containing a solution of compound **4b** (540 mg, 2.4 mmol) in CH_2_Cl_2_ (10 mL) was cooled in an ice bath, and sulfuryl chloride (647 mg, 4.8 mmol) was added dropwise under nitrogen. After 1h, the reaction mixture was concentrated under reduced pressure to afford the product as a colorless oil. Yield: 470 mg, 93%; ν_max_ (liquid)/cm^–1^ 2918 (C–H), 1700 (C=O), 1055 (C–O), 837 (C–Cl); δ_H_ (400 MHz; CDCl_3_) 7.66 (d, *J* = 9.5 Hz, 1H), 7.46 (d, *J* = 8.7 Hz, 1H), 7.07 (d, *J* = 2.6 Hz, 1H), 7.01 (dd, *J* = 8.6 and 2.4 Hz, 1H), 6.33 (d, *J* = 9.5 Hz, 1H), 5.91 (s, 2H); δ_C_ (101 MHz; CDCl_3_) 160.76 (C=O), 158.41 (Ar C–O), 155.57 (Ar C–O), 143.14 (C=C), 129.18 (Ar C–H), 114.87 (Ar C–H), 114.64 (Ar C–H), 113.43 (C=C), 103.89 (Ar C–H), 76.09 (Cl–C–O); *m*/*z* (APCI^+^) 211 ([M + H]^+^, 100%); HRMS calculated for C_10_H_7_ClO_3_ [M + H]^+^ 211.0156, found 211.0162, δ 2.6 ppm.

#### 4.5.6 7-(((*tert*-Butyldimethylsilyl)oxy)methoxy)-2*H*-chromen-2-one, **4**

Compound **4c** (470 mg, 2 mmol) was dissolved in anhydrous DMF (5 mL) under a nitrogen atmosphere, and then sodium hydroxide (160 mg, 4 mmol) was added. The resulting solution was stirred for 20 min, and the *tert*-butyldimethylsilanol (0.3 mL, 2 mmol) as a solution in anhydrous DMF (5 mL) was added dropwise over a period of 15 min. The reaction mixture was stirred overnight, and the mixture was poured into water (50 mL) and extracted with ethyl acetate (3 × 40 mL). The combined organic layers were dried with MgSO_4_ and concentrated under reduced pressure. The crude product was purified by column chromatography (hexane/ethyl acetate, 3:2) to afford the desired product as white crystals. Yield: 209 mg, 34%; *R*_f_ 0.52 (hexane/ethyl acetate = 3:2); ν_max_ (solid)/cm^–1^ 2856 (C–H), 1708 (C=O), 1075 (C–O), 997 (C=C), 830 (Si–O); δ_H_ (400 MHz; CDCl_3_) 7.64 (d, *J* = 9.5 Hz, 1H), 7.37 (d, *J* = 8.6 Hz, 1H), 7.01 (d, *J* = 1.8 Hz, 1H), 6.93 (dd, *J* = 8.6 and 1.7 Hz, 1H), 6.26 (d, *J* = 9.5 Hz, 1H), 5.43 (s, 2H), 0.88 (s, 9H), 0.12 (s, 6 H); δ_C_ (101 MHz; CDCl_3_) 161.70 (C=O), 161.12 (Ar C–O), 156.12 (Ar C–O), 143.84 (C=C), 129.13 (Ar C–H), 114.05 (Ar C–C), 113.95 (Ar C–H), 113.60 (C=C), 103.91 (Ar C–H), 88.39 (O–C–O), 26.00 (Si–C–CH_3_), 18.35 (Si–C–CH_3_), −4.61 (Si–CH_3_); *m*/*z* (APCI ^+^) 307 ([M + H]^+^, 100%); HRMS calculated for C_16_H_22_O_4_Si [M + H]^+^ 307.1360, found 307.1374, δ 4.5 ppm.

## Supporting Information Available

Concentration of phenoxides produced due to uncatalyzed and catalyzed hydrolysis of substrates **1**–**4** (50 and 100 μM); table of initial rates of enzyme-catalyzed reaction and the background hydrolysis of substrates **1**–**4** (50 and 100 μM); table of the average concentration of **1a** formed due to catalyzed reactions, the standard deviation of triplicate results and the calculated *Z*-factor value; graph of concentration of corresponding phenoxide products formed due to TF-Silα-Strep catalyzed reactions with varying amounts of starting substrate concentration; Michaelis–Menten curves for the hydrolysis of substrates by TF-Silα-Strep against a range of concentrations; image of SDS-PAGE gel of isolated TF-Silα-Strep; calibration curve of the fluorescence signal against known concentration of the corresponding phenoxides; image of the black 96-well microtiter plate containing varying concentrations of product **1a** in Tris Buffer used to calculate the calibration curve; schemes showing the synthetic routes for **2** and **4**; ^1^H and ^13^C NMR spectra for synthesized substrates; and mass spectra of synthesized substrates (PDF)
