# Supplementary material for: Evaluation of Fluorescence-Based Screening Assays for the Detection and Quantification of Silyl Hydrolase Activity
Source: ACS Omega. 2024 Jun 25;9(27):29939–46. doi: 10.1021/acsomega.4c05409 (PMC11238280; doi:10.1021/acsomega.4c05409)
Supplement: Supplementary file 1 — ao4c05409_si_001.pdf [file ao4c05409_si_001.pdf]

Supplementary Information

Evaluation of fluorescence–based screening assays for the detection and quantification of silyl hydrolase activity

Jason Z. He,[a,b] Yuqing Lu,[a,b] Neha Jain,[c] David G. Churchill,[c]* Lu Shin Wong[a,b]*

[a] Manchester Institute of Biotechnology, The University of Manchester, 131 Princess Street, Manchester M1 7DN, United Kingdom.

[b] Department of Chemistry, The University of Manchester, Oxford Road, Manchester M13 9PL, United Kingdom.

[c] Department of Chemistry, Korea Advanced Institute of Science and Technology, Daejeon, 34141, Republic of Korea

* Correspondence: l.s.wong@manchester.ac.uk, dchurchill@kaist.ac.kr

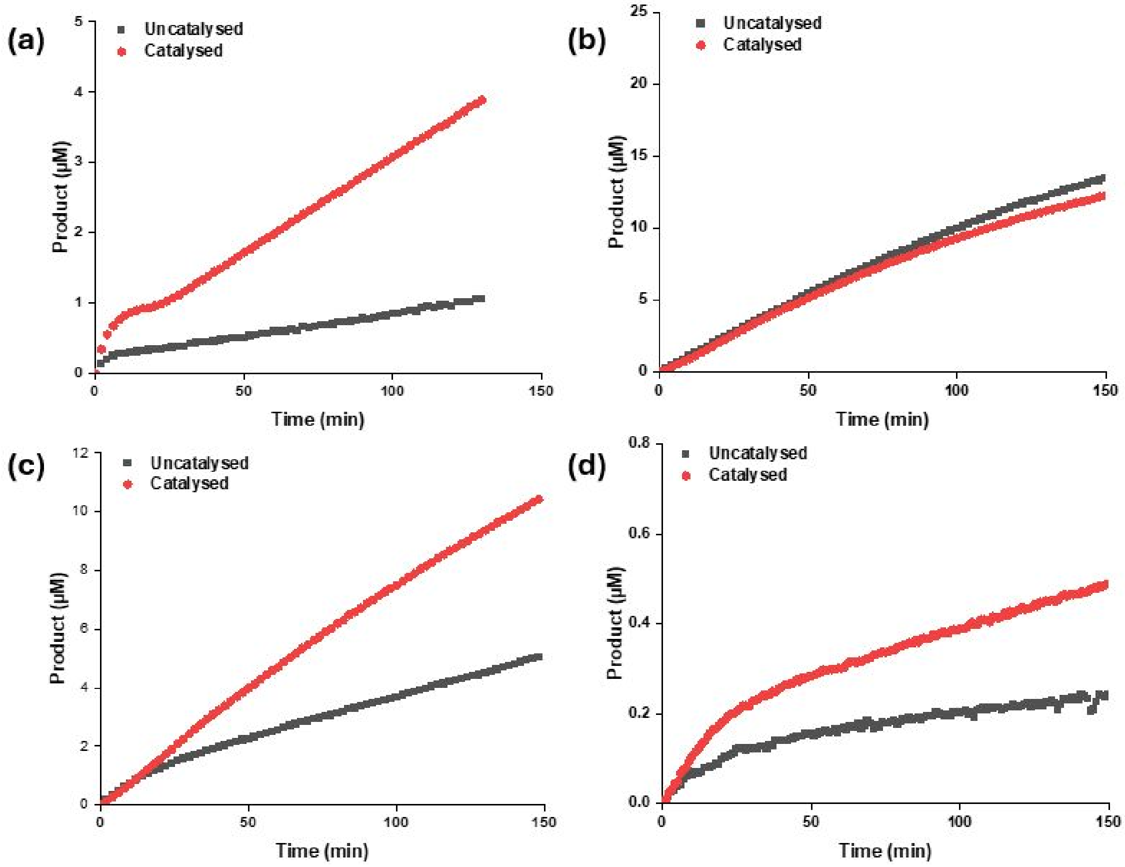

Figure S1. Graphs of average product concentrations against time for the uncatalyzed and catalyzed hydrolysis of substrates **1-4** (a-d respectively) at 50 μM substrate concentration. Reactions were carried out with 6.7 μM TF-Silα-Strep, 10 % v/v 1,4-dioxane, 50 mM Tris buffer and 100 mM NaCl at pH 8.5. The enzyme was omitted for the uncatalyzed control reactions.

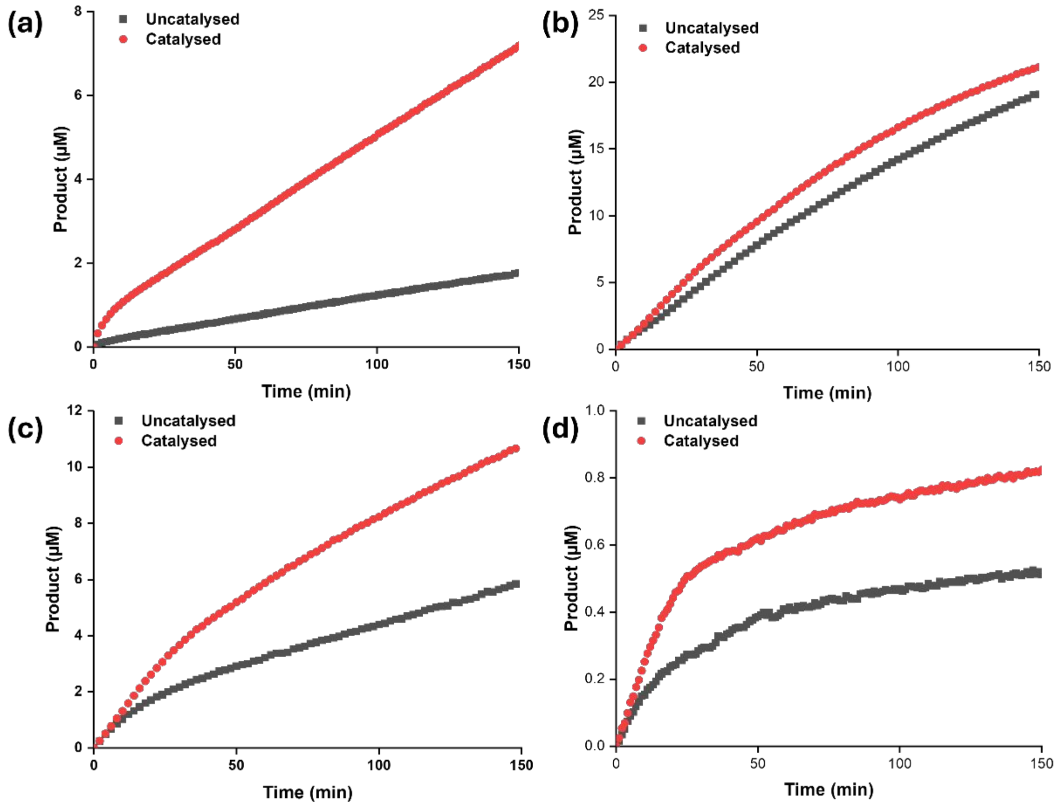

Figure S2. Graphs of average product concentrations against time for the uncatalyzed and catalyzed hydrolysis of substrates **1-4** (a-d respectively) at 100 µM substrate concentration. Reactions were carried out with 6.7 µM TF-Silα-Strep, 10 % v/v 1,4-dioxane, 50 mM Tris buffer and 100 mM NaCl at pH 8.5. The enzyme was omitted for the uncatalyzed control reactions.

Table S1. Table of initial rates of enzyme catalyzed reaction and the background hydrolysis of substrates **1-4**. Enzymatic reactions were carried out with 6.7 μM TF-Silα-Strep, 50 or 100 μM substrate, 10 % v/v 1,4-dioxane, 50 mM Tris buffer at pH 8.5 and 100 mM NaCl.

**Substrate**	**Initial rate at 50 μM substrate (μM min^{-1})**					
	**Uncatalyzed**	SEM	**Catalyzed**	SEM	**Net***	SEM
**1**	0.0359	0.0048	0.0977	0.0114	0.0618	0.0123
**2**	0.1085	0.0009	0.1099	0.0004	0.0014	0.0010
**3**	0.0646	0.0018	0.0818	0.0007	0.0172	0.0019
**4**	0.0059	0.0004	0.0106	0.0002	0.0047	0.0004
	**Initial rate at 100 μM substrate (μM min^{-1})**					
**1**	0.0319	0.0055	0.1299	0.0120	0.0980	0.0132
**2**	0.1569	0.0008	0.2127	0.0017	0.0558	0.0018
**3**	0.0841	0.0034	0.1267	0.0015	0.0426	0.0037
**4**	0.0160	0.0005	0.0251	0.0003	0.0091	0.0006

*Net rate calculated as the difference between the catalyzed rate value and the uncatalyzed rate value.

Table S2. Table of average concentration of **1a** formed due to catalyzed reactions, the standard deviation of triplicate results and the calculated Z-factor value.

Time (min)	Cell lysates with TF-Silα-Strep (μM)	σ	Cell lysates without TF-Silα-Strep (μM)	σ	**Z-Factor***
0	0.000	0.000	0.000	0.000	**0.00**
2	0.317	0.188	0.068	0.239	**-4.14**
4	0.489	0.211	0.293	0.249	**-6.03**
6	0.748	0.215	0.431	0.282	**-3.69**
8	0.968	0.183	0.569	0.253	**-2.28**
10	1.111	0.150	0.607	0.212	**-1.16**
12	1.231	0.127	0.624	0.215	**-0.69**
14	1.250	0.109	0.659	0.236	**-0.76**
16	1.256	0.129	0.686	0.222	**-0.85**
18	1.317	0.138	0.710	0.217	**-0.76**
20	1.358	0.104	0.747	0.223	**-0.60**
22	1.387	0.087	0.783	0.230	**-0.57**
24	1.397	0.120	0.810	0.234	**-0.81**
26	1.448	0.122	0.827	0.230	**-0.70**
28	1.504	0.127	0.870	0.220	**-0.64**
30	1.559	0.124	0.879	0.218	**-0.51**
32	1.608	0.120	0.934	0.230	**-0.56**
34	1.647	0.121	0.960	0.230	**-0.53**
36	1.702	0.116	0.983	0.227	**-0.43**
38	1.765	0.125	1.020	0.230	**-0.43**
40	1.809	0.128	1.051	0.243	**-0.47**
42	1.855	0.121	1.072	0.244	**-0.40**
44	1.901	0.133	1.110	0.231	**-0.38**
46	1.950	0.127	1.130	0.240	**-0.34**
48	2.002	0.116	1.163	0.236	**-0.26**
50	2.050	0.120	1.187	0.245	**-0.27**
52	2.082	0.133	1.222	0.232	**-0.27**
54	2.140	0.125	1.244	0.232	**-0.20**
56	2.188	0.120	1.266	0.238	**-0.16**
58	2.230	0.139	1.305	0.240	**-0.23**
60	2.282	0.129	1.338	0.236	**-0.16**
62	2.326	0.113	1.368	0.247	**-0.13**
64	2.382	0.119	1.405	0.241	**-0.10**
66	2.437	0.124	1.433	0.242	**-0.09**

68	2.477	0.135	1.468	0.241	**-0.12**
70	2.519	0.121	1.491	0.245	**-0.07**
72	2.567	0.135	1.519	0.251	**-0.10**
74	2.617	0.123	1.549	0.242	**-0.03**
76	2.669	0.122	1.585	0.237	**0.00**
78	2.713	0.130	1.604	0.239	**0.00**
80	2.750	0.142	1.640	0.260	**-0.09**
82	2.807	0.134	1.683	0.247	**-0.02**
84	2.856	0.128	1.707	0.251	**0.01**
86	2.888	0.125	1.731	0.262	**0.00**
88	2.933	0.126	1.763	0.251	**0.03**
90	2.977	0.130	1.809	0.255	**0.01**
92	3.020	0.119	1.831	0.255	**0.06**
94	3.065	0.137	1.845	0.232	**0.09**
96	3.113	0.119	1.874	0.249	**0.11**
98	3.157	0.126	1.916	0.266	**0.05**
100	3.199	0.119	1.954	0.267	**0.07**
102	3.233	0.127	1.973	0.262	**0.08**
104	3.294	0.136	2.006	0.274	**0.05**
106	3.333	0.146	2.042	0.270	**0.03**
108	3.377	0.132	2.072	0.271	**0.07**
110	3.416	0.138	2.108	0.254	**0.10**
112	3.461	0.128	2.124	0.264	**0.12**
114	3.513	0.137	2.148	0.254	**0.14**
116	3.539	0.142	2.193	0.246	**0.14**
118	3.591	0.120	2.204	0.273	**0.15**
120	3.647	0.141	2.263	0.268	**0.11**
122	3.688	0.133	2.288	0.250	**0.18**
124	3.724	0.127	2.318	0.269	**0.16**
126	3.769	0.124	2.350	0.258	**0.19**
128	3.822	0.132	2.376	0.270	**0.17**
130	3.862	0.120	2.396	0.268	**0.21**
132	3.896	0.146	2.430	0.279	**0.13**
134	3.937	0.116	2.466	0.274	**0.20**
136	3.994	0.120	2.488	0.283	**0.20**
138	4.028	0.118	2.536	0.273	**0.21**
140	4.052	0.139	2.545	0.253	**0.22**
142	4.099	0.142	2.582	0.273	**0.18**
144	4.138	0.124	2.628	0.267	**0.22**
146	4.193	0.123	2.645	0.275	**0.23**
148	4.237	0.137	2.677	0.266	**0.22**
150	4.284	0.146	2.716	0.274	**0.20**
152	4.319	0.116	2.734	0.301	**0.21**
154	4.359	0.137	2.759	0.280	**0.22**

156	4.394	0.097	2.790	0.277	**0.30**
158	4.450	0.136	2.831	0.277	**0.23**
160	4.485	0.145	2.863	0.278	**0.22**
162	4.532	0.132	2.887	0.285	**0.24**
164	4.575	0.124	2.924	0.270	**0.28**
166	4.610	0.141	2.931	0.279	**0.25**
168	4.649	0.112	2.978	0.286	**0.29**
170	4.690	0.100	3.011	0.301	**0.28**
172	4.742	0.097	3.032	0.281	**0.34**
174	4.779	0.092	3.071	0.282	**0.34**
176	4.817	0.113	3.103	0.284	**0.31**
178	4.858	0.118	3.140	0.272	**0.32**
180	4.892	0.141	3.165	0.280	**0.27**
182	4.941	0.122	3.207	0.290	**0.29**
184	4.977	0.152	3.228	0.279	**0.26**
186	5.011	0.139	3.252	0.290	**0.27**
188	5.069	0.119	3.280	0.285	**0.32**
190	5.084	0.135	3.313	0.272	**0.31**
192	5.140	0.118	3.338	0.282	**0.33**
194	5.186	0.118	3.395	0.283	**0.33**
196	5.230	0.118	3.409	0.296	**0.32**
198	5.248	0.129	3.432	0.298	**0.29**
200	5.324	0.115	3.468	0.281	**0.36**
202	5.338	0.108	3.502	0.317	**0.31**
204	5.370	0.128	3.497	0.295	**0.32**
206	5.392	0.154	3.572	0.294	**0.26**
208	5.470	0.108	3.587	0.290	**0.37**
210	5.495	0.127	3.615	0.301	**0.32**
212	5.563	0.128	3.649	0.289	**0.35**
214	5.595	0.112	3.672	0.326	**0.32**
216	5.647	0.110	3.735	0.324	**0.32**
218	5.657	0.111	3.725	0.302	**0.36**
220	5.713	0.125	3.769	0.317	**0.32**
222	5.744	0.102	3.795	0.305	**0.37**
224	5.773	0.091	3.828	0.310	**0.38**
226	5.842	0.106	3.854	0.297	**0.39**
228	5.859	0.114	3.890	0.302	**0.37**
230	5.921	0.097	3.910	0.320	**0.38**
232	5.949	0.128	3.945	0.321	**0.33**
234	5.993	0.134	3.966	0.318	**0.33**
236	5.988	0.132	4.026	0.298	**0.34**
238	6.059	0.115	4.041	0.313	**0.36**
240	6.113	0.107	4.081	0.319	**0.37**
242	6.131	0.124	4.093	0.325	**0.34**

244	6.180	0.102	4.143	0.332	**0.36**
246	6.190	0.098	4.179	0.312	**0.39**
248	6.254	0.101	4.193	0.333	**0.37**
250	6.316	0.136	4.239	0.314	**0.35**
252	6.344	0.114	4.263	0.290	**0.42**
254	6.414	0.116	4.255	0.288	**0.44**
256	6.424	0.103	4.318	0.305	**0.42**
258	6.452	0.122	4.335	0.317	**0.38**
260	6.475	0.151	4.366	0.331	**0.31**
262	6.545	0.126	4.402	0.319	**0.38**
264	6.542	0.115	4.416	0.325	**0.38**
266	6.648	0.102	4.440	0.324	**0.42**
268	6.709	0.094	4.469	0.306	**0.46**
270	6.738	0.126	4.525	0.335	**0.37**

*Z-factor $= 1 - \frac{(3\sigma_s + 3\sigma_c)}{|\mu_s - \mu_c|}$

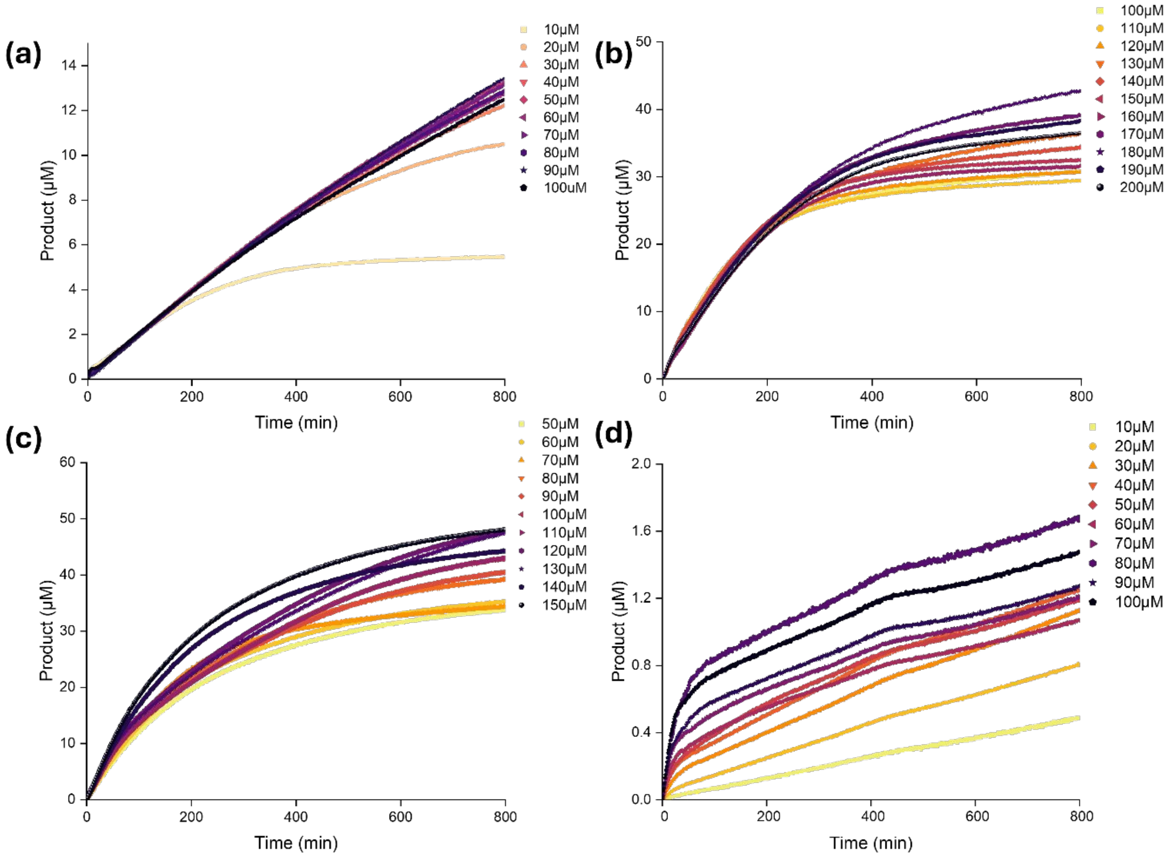

Figure S3. Graphs of concentration of corresponding phenoxide products formed due to TF-Silα-Strep catalyzed reactions with varying amounts of starting substrate concentration as measured by fluorescence of their excitation and emission wavelengths. Graph (a) represents substrate **1**, (b) for **2**, (c) for **3**, and (d) for **4**.

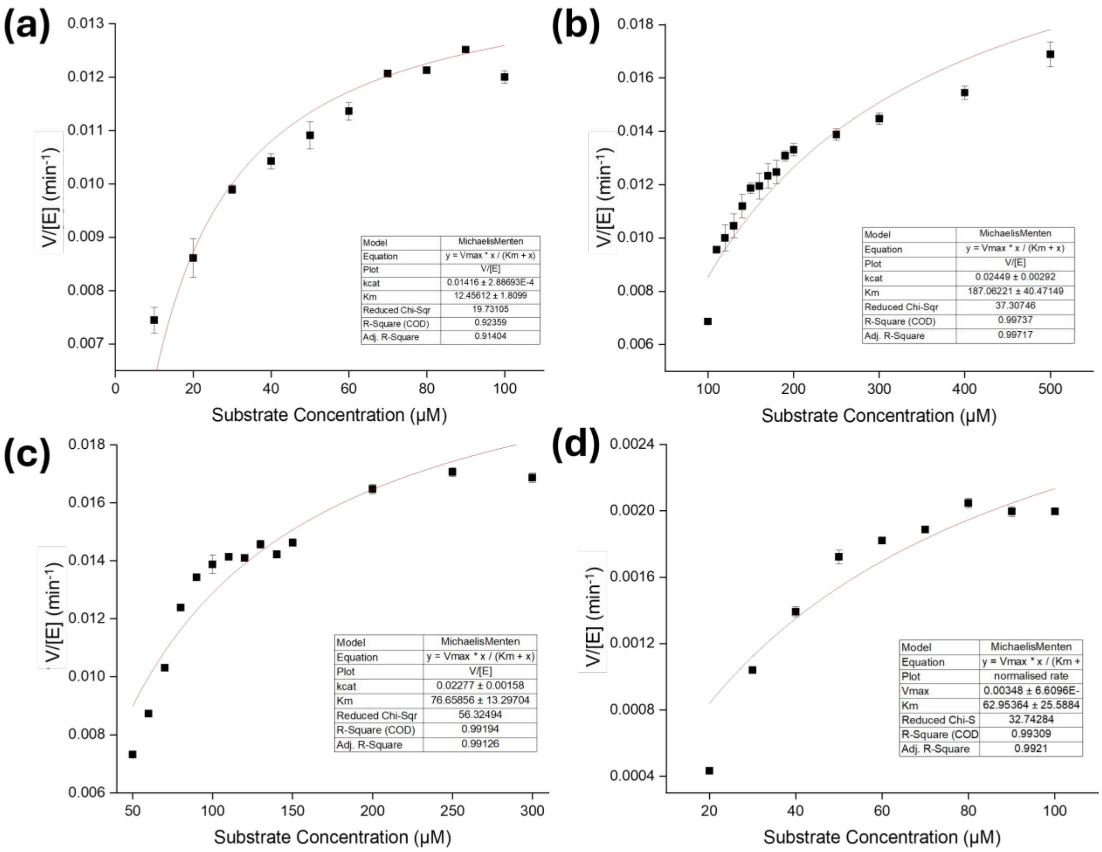

Figure S4. Michaelis-Menten curves for the hydrolysis of substrates by TF-Silα-Strep against a range of concentrations. Graph (a) represents substrate **1**, (b) for **2**, (c) for **3**, and (d) for **4**.

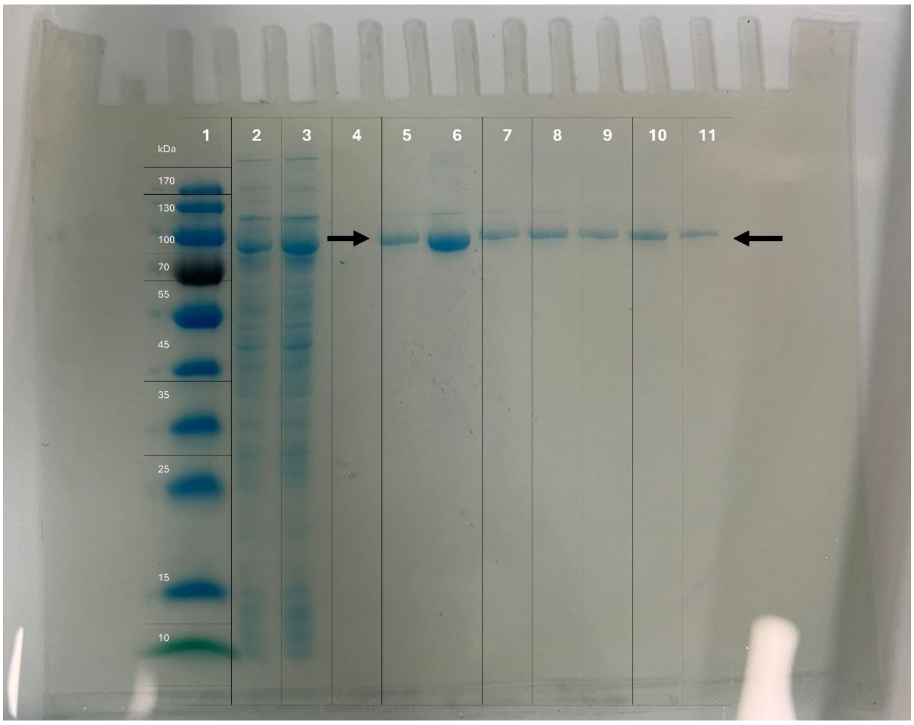

Figure S5. Image of SDS-PAGE gel of TF-Silα-Strep. Lane 1 shows the prestained protein ladder, lanes 2-3 show the unpurified fractions of the protein and lanes 5-11 show purified fractions of the protein from the FPLC. Arrows indicate the isolation and presence of the desired protein mark

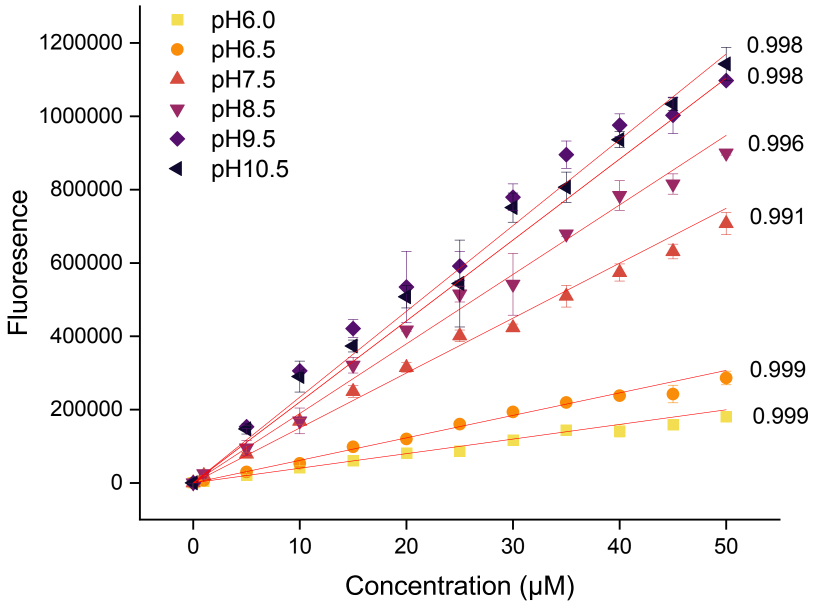

Figure S6. Calibration curve of fluorescence signal against concentration of **1a.** Solutions prepared in the same buffer used for the enzyme assays: 10% v/v 1,4-dioxane, 50mM Tris buffer and 100mM NaCl. R-squared values of the line of best fit are shown to the right.

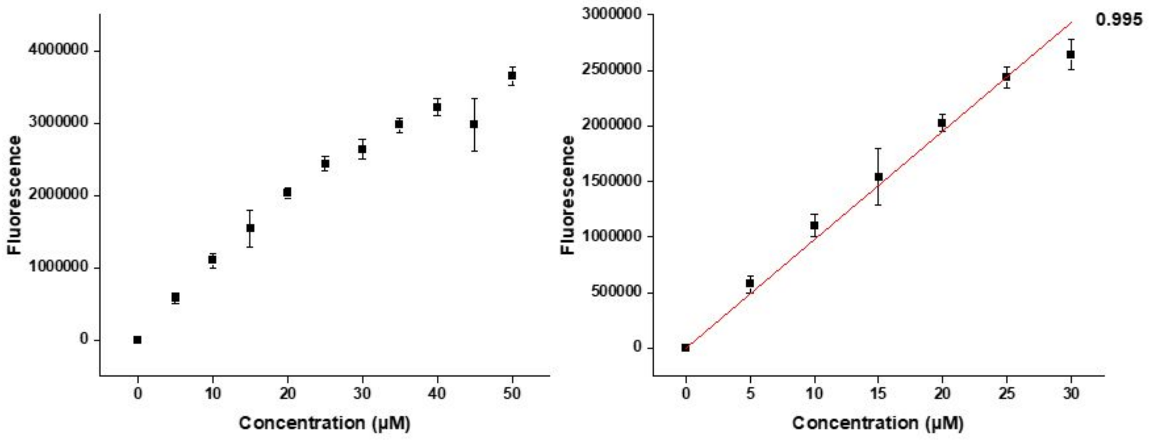

Figure S7. Plots of fluorescence signal against concentration of **2a**. The graph on the left shows signals over a larger range of concentration. The graph on the right shows fitting taken from the most linear range at lower concentrations. R-squared value of the line of best fit is shown to the right.

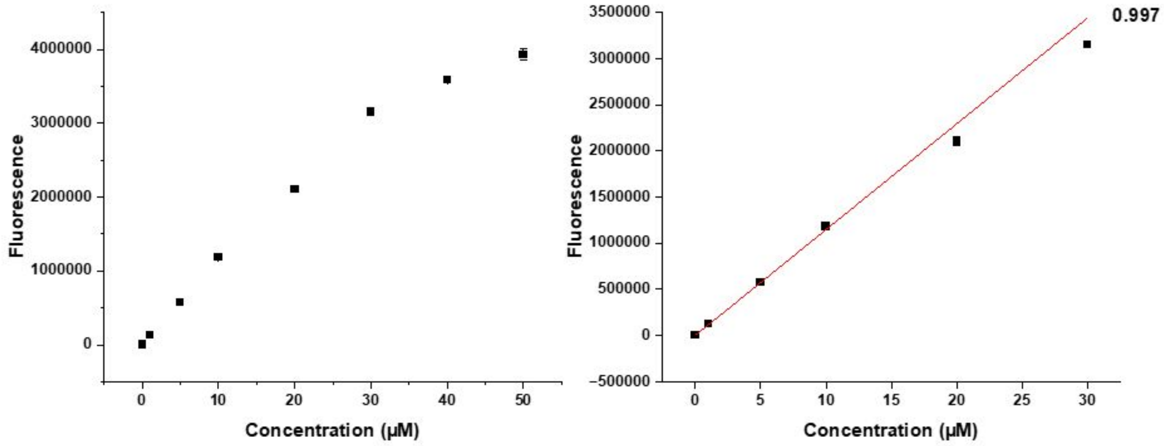

Figure S8. Plots of fluorescence signal against concentration of **3a**. The graph on the left shows signals over a larger range of concentration. The graph on the right shows fitting taken from the most linear range at lower concentrations. R-squared value of the line of best fit is shown to the right.

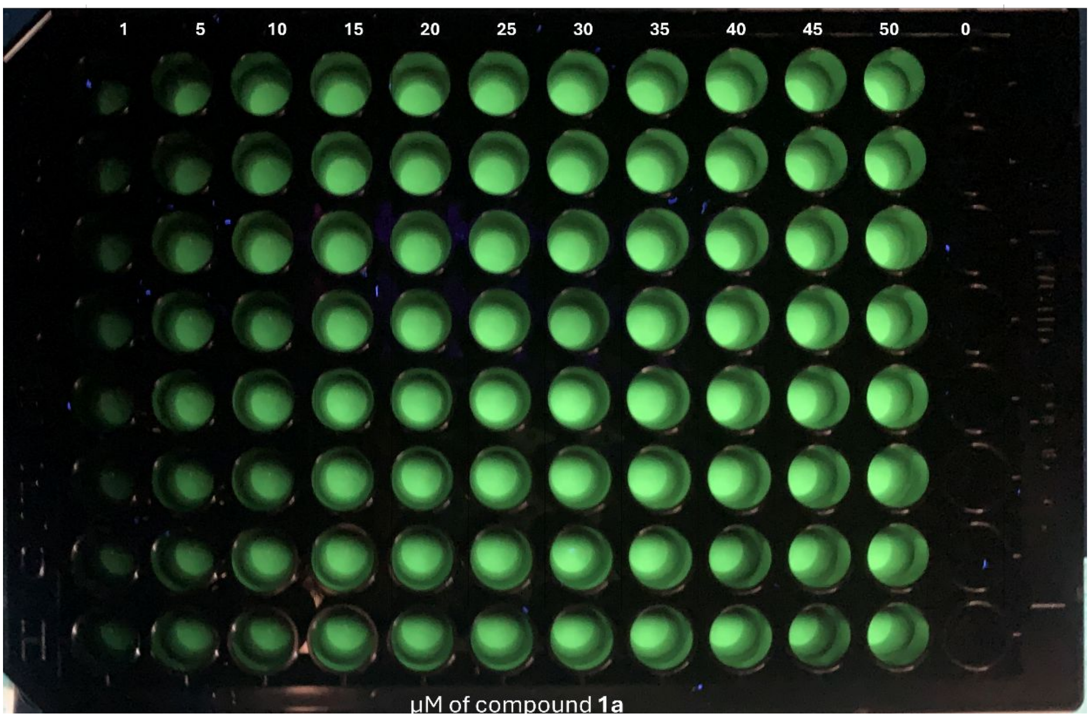

Figure S9. Image of microtitre plate under UV lamp containing varying concentrations of **1a** in Tris Buffer (50 µM Tris, 100 µM NaCl at pH 8.5) used to calculate the calibration curve.

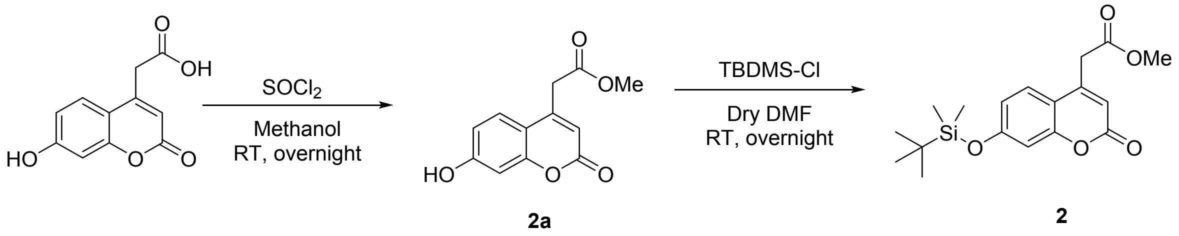

Scheme S1. Synthesis of substrate **2.**

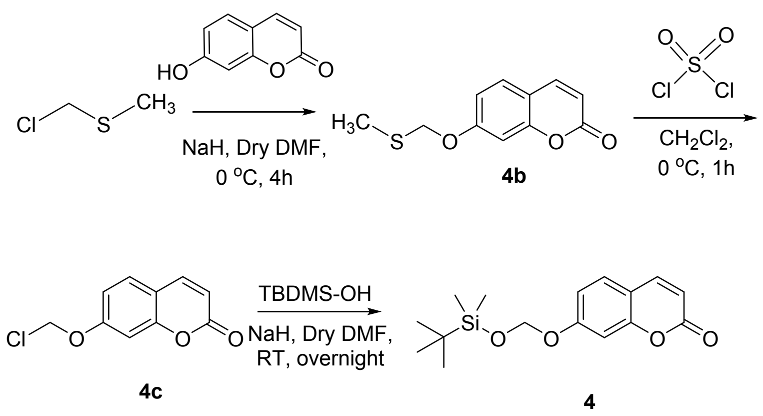

Scheme S2. Synthesis of substrate **4**.

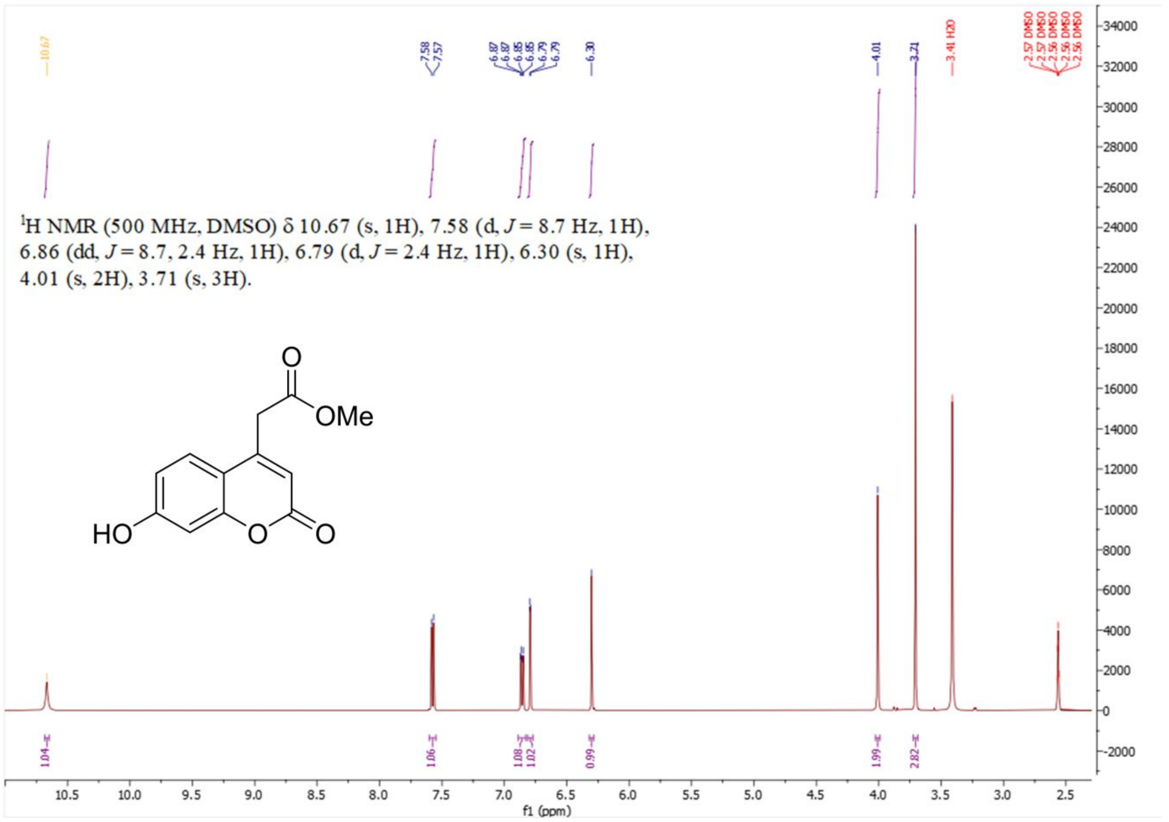

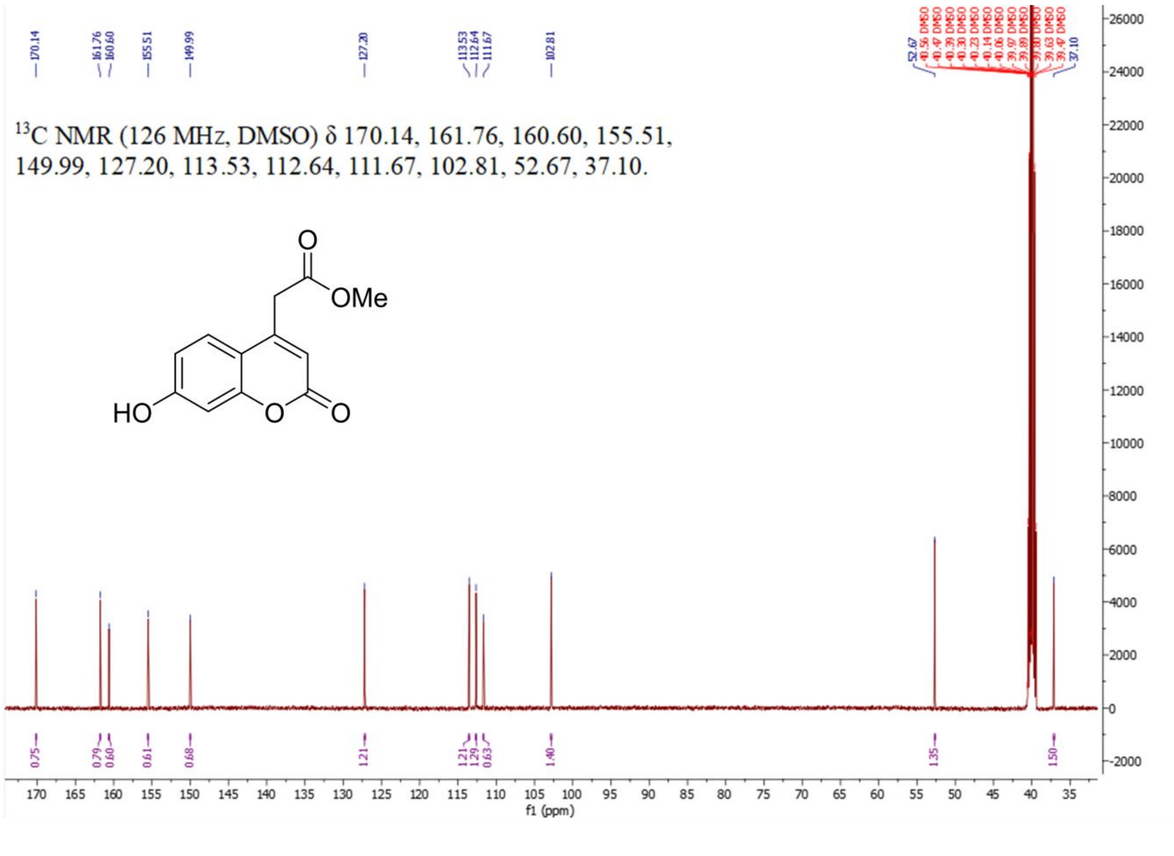

Figure S10. Calibrated NMR Spectra of **2a** showing ^{1}H (**top**) and ^{13}C (**bottom**) chemical shifts.

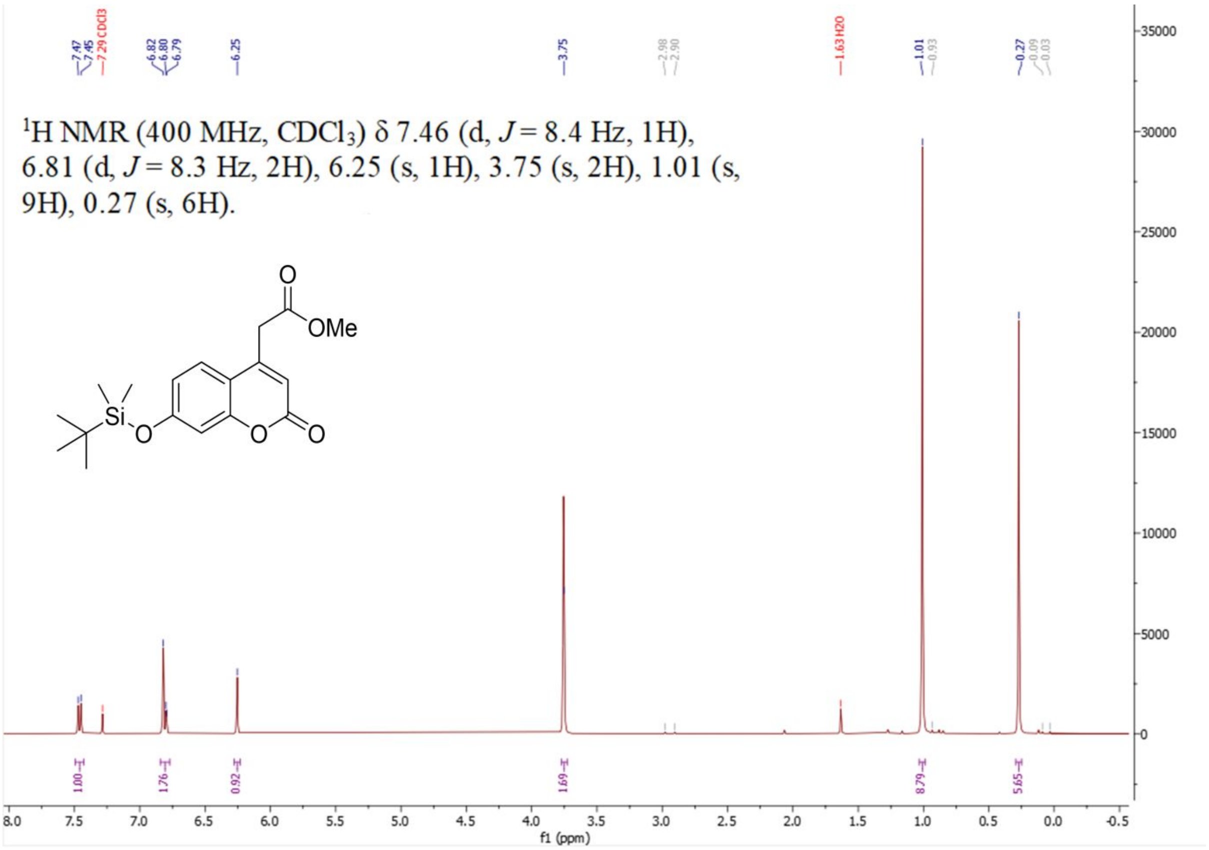

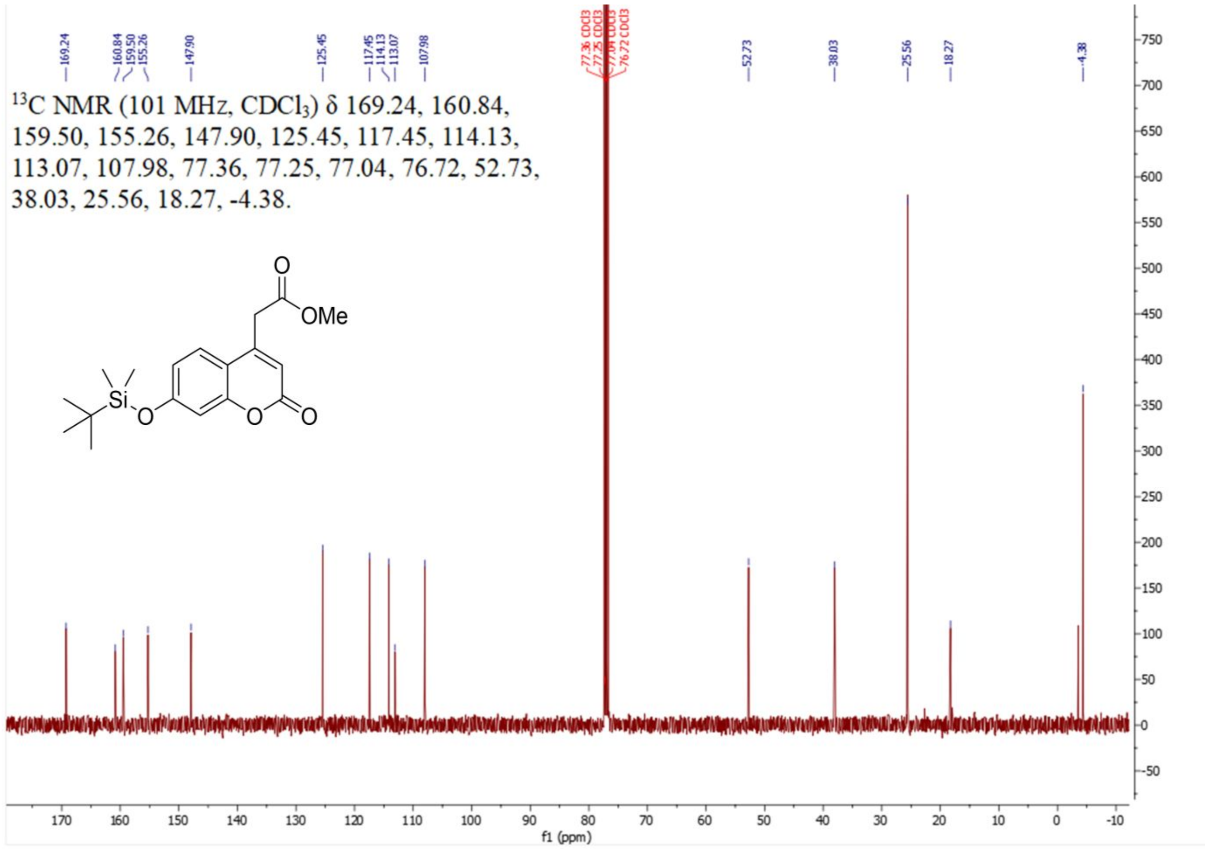

Figure S11. Calibrated NMR Spectra of **2** showing ^{1}H (**top**) and ^{13}C (**bottom**) chemical shifts.

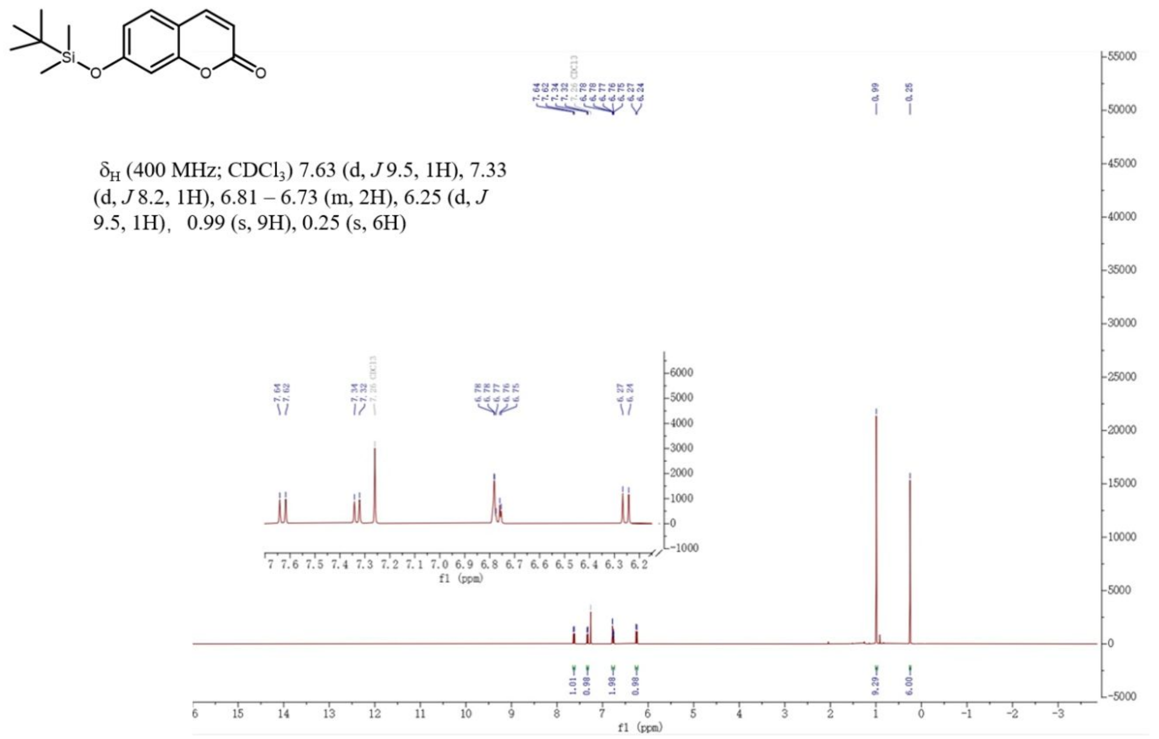

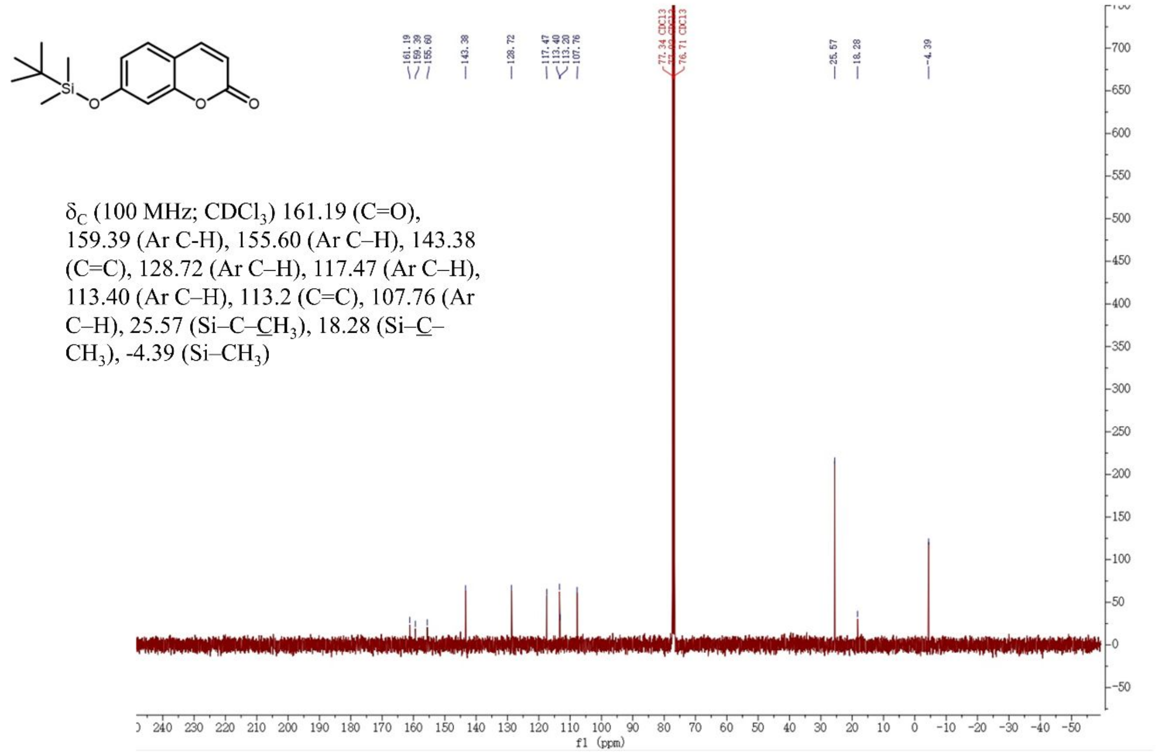

Figure S12. Calibrated NMR Spectra of **3** showing ^{1}H (**top**) and ^{13}C (**bottom**) chemical shifts.

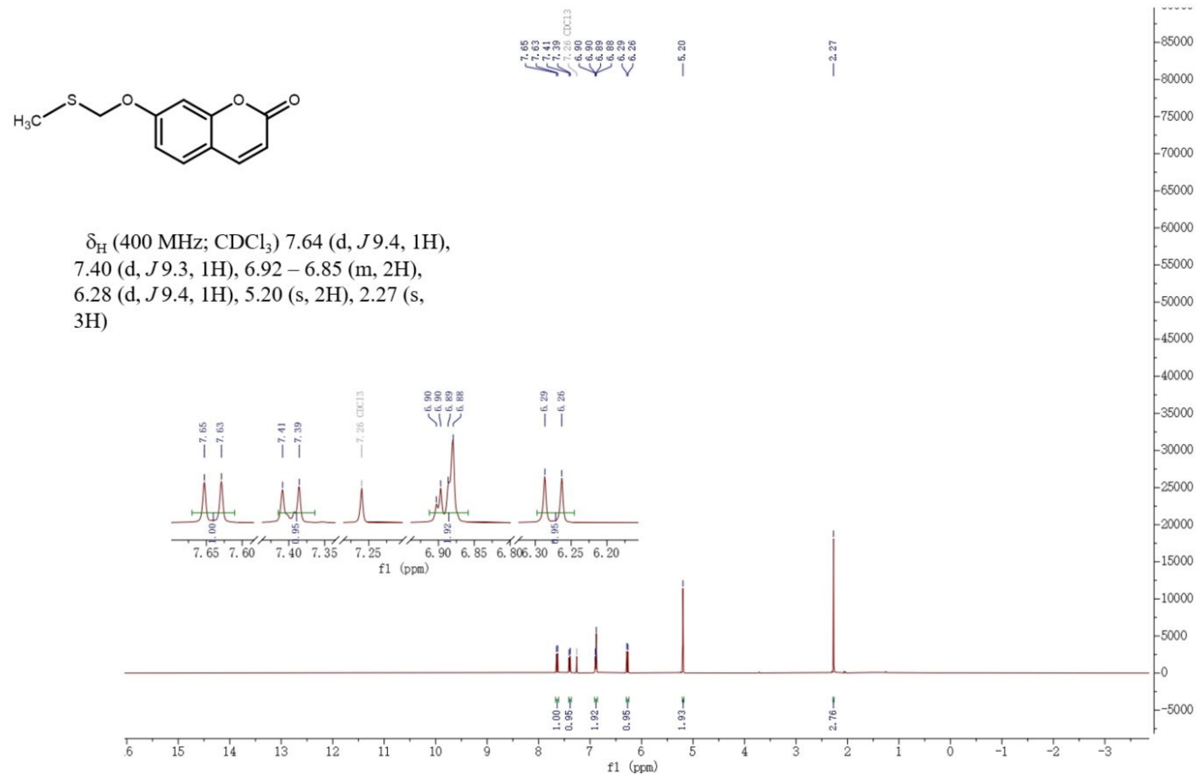

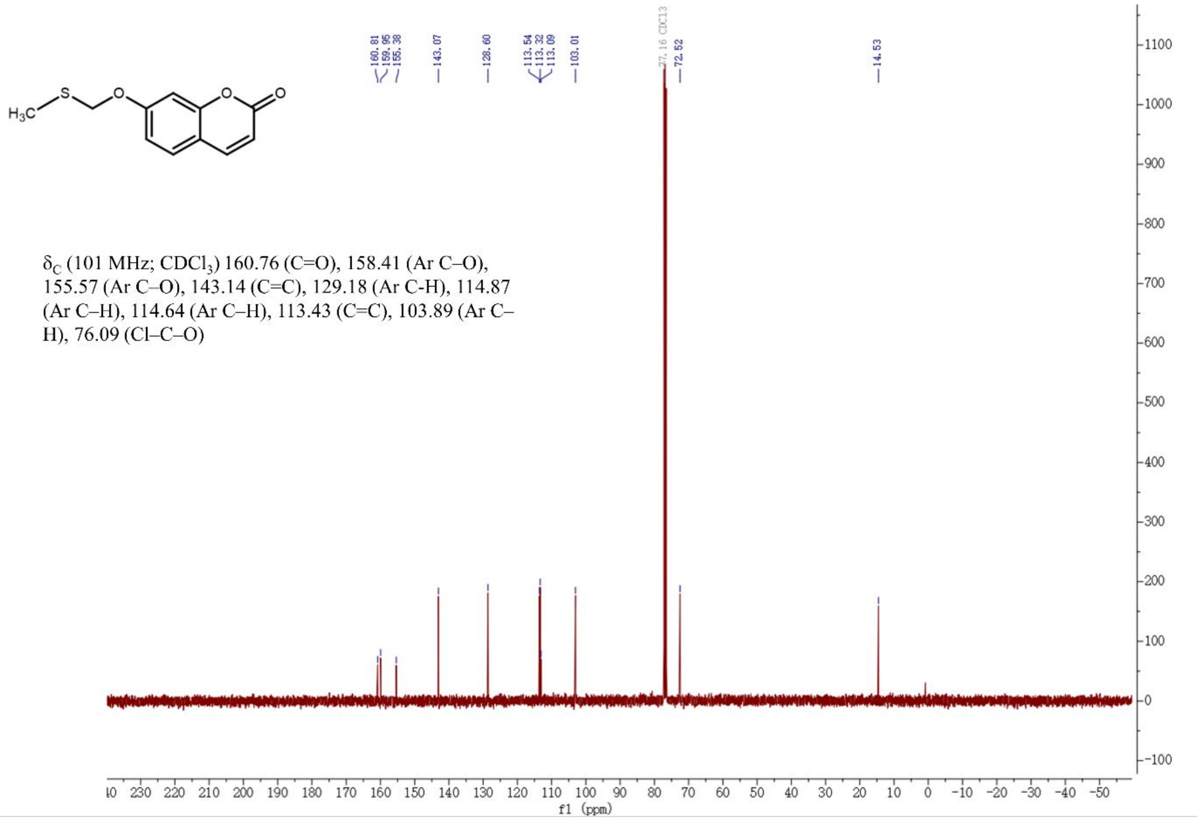

Figure S13. Calibrated NMR spectra of **4b** showing ^{1}H (**top**) and ^{13}C (**bottom**) chemical shifts.

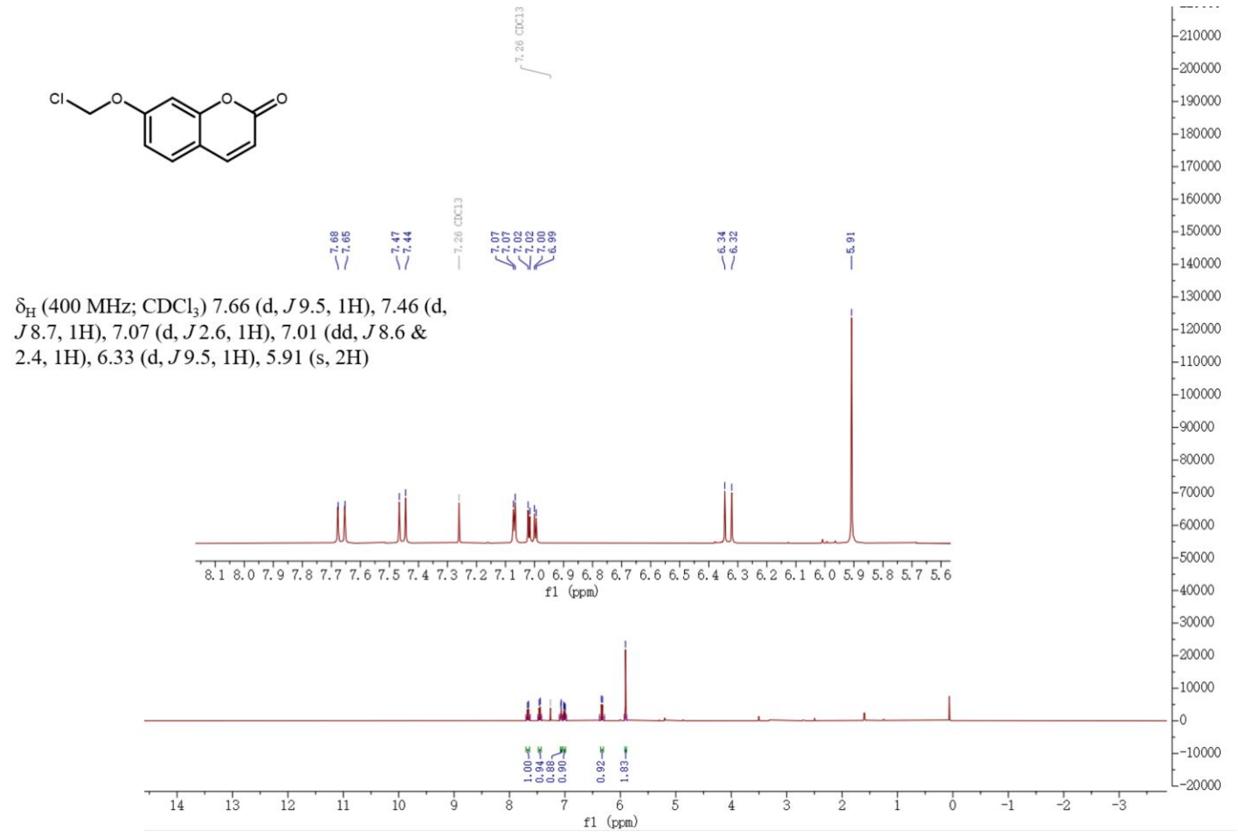

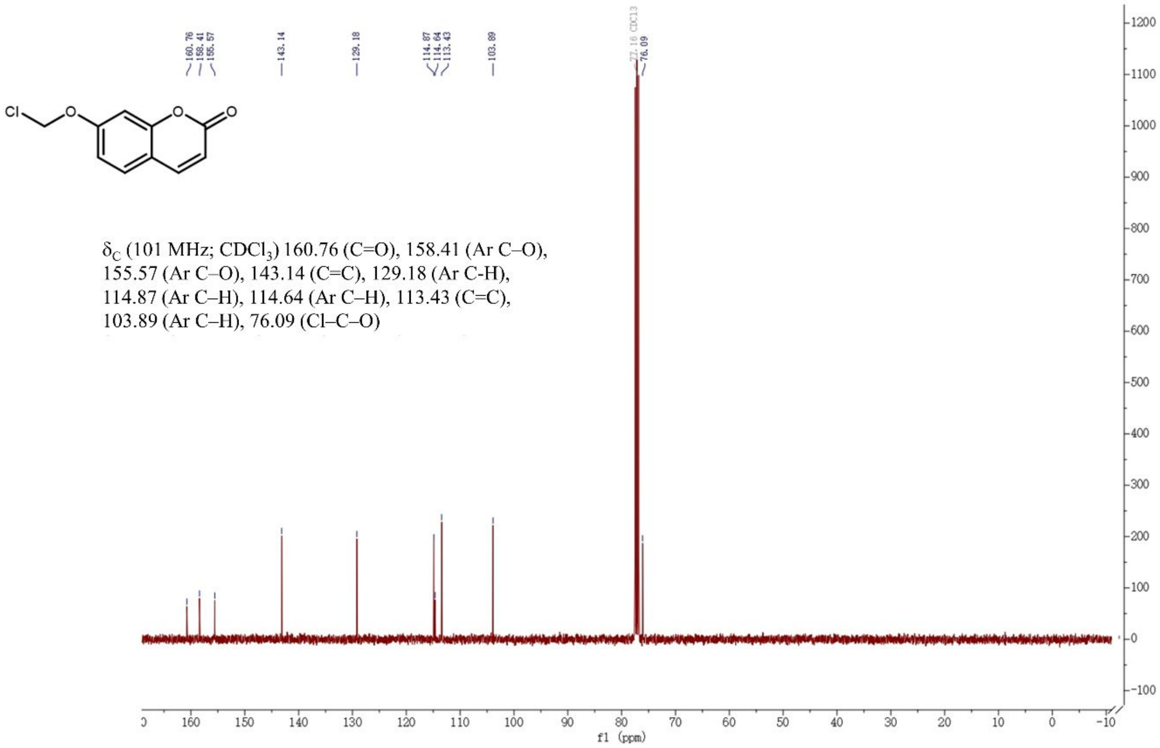

Figure S14. Calibrated NMR spectra of **4c** showing ^{1}H (**top**) and ^{13}C (**bottom**) chemical shifts.

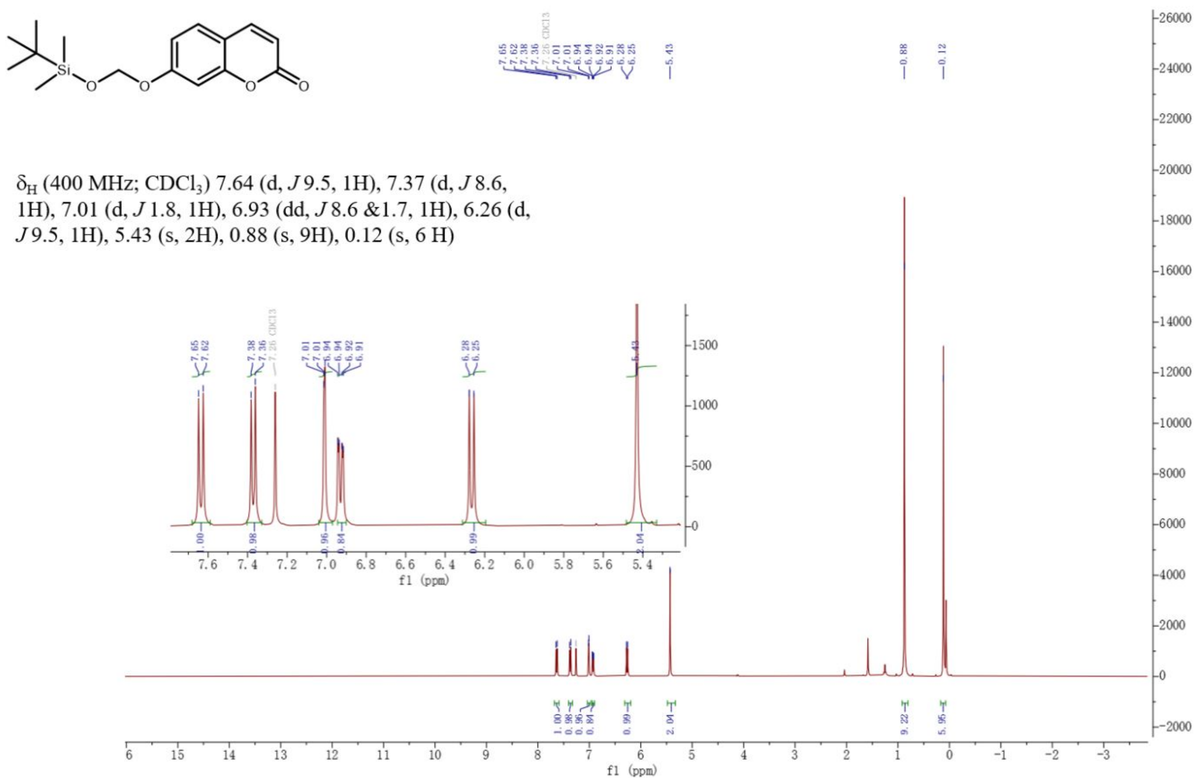

δ_H (400 MHz; $CDCl_3$) 7.64 (d, *J* 9.5, 1H), 7.37 (d, *J* 8.6, 1H), 7.01 (d, *J* 1.8, 1H), 6.93 (dd, *J* 8.6 &1.7, 1H), 6.26 (d, *J* 9.5, 1H), 5.43 (s, 2H), 0.88 (s, 9H), 0.12 (s, 6 H)

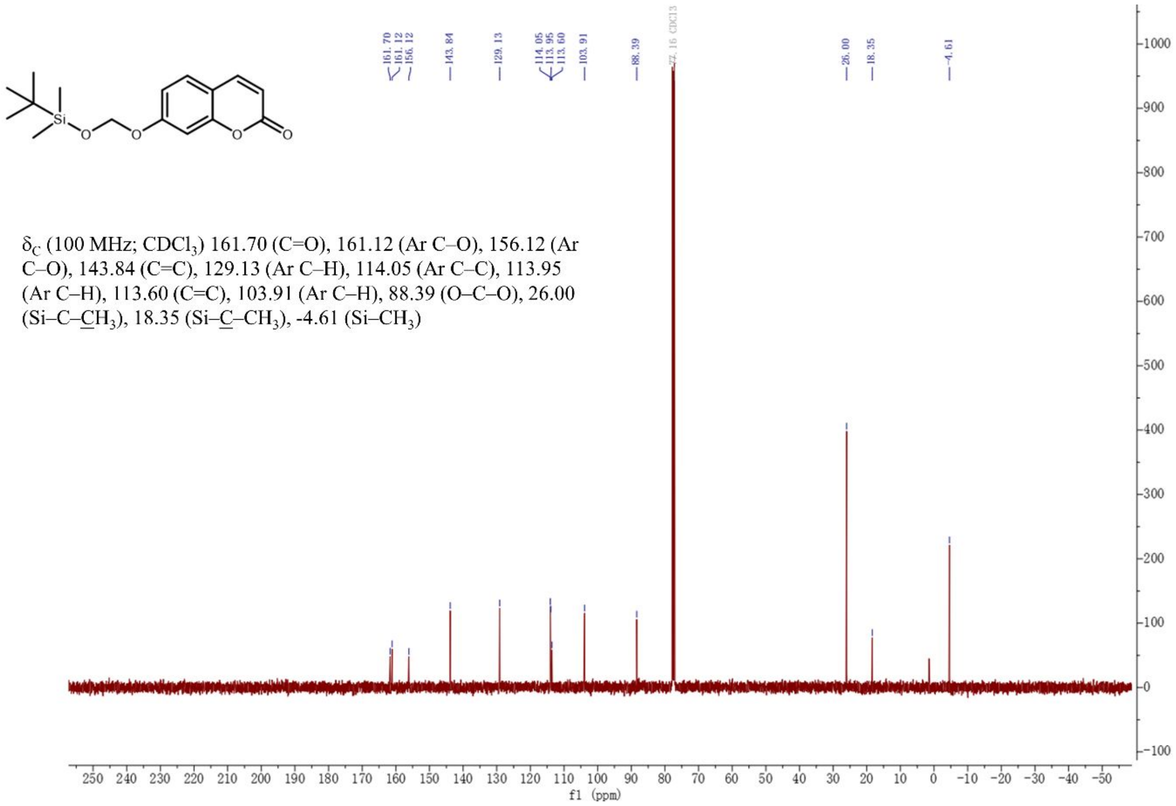

δ_C (100 MHz; $CDCl_3$) 161.70 (C=O), 161.12 (Ar C–O), 156.12 (Ar C–O), 143.84 (C=C), 129.13 (Ar C–H), 114.05 (Ar C–C), 113.95 (Ar C–H), 113.60 (C=C), 103.91 (Ar C–H), 88.39 (O–C–O), 26.00 (Si–C–$\underline{C}H_3$), 18.35 (Si–$\underline{C}$–CH_3), -4.61 (Si–CH_3)

Figure S15. Calibrated NMR spectra of **4** showing ^{1}H (**top**) and ^{13}C (**bottom**) chemical shifts.

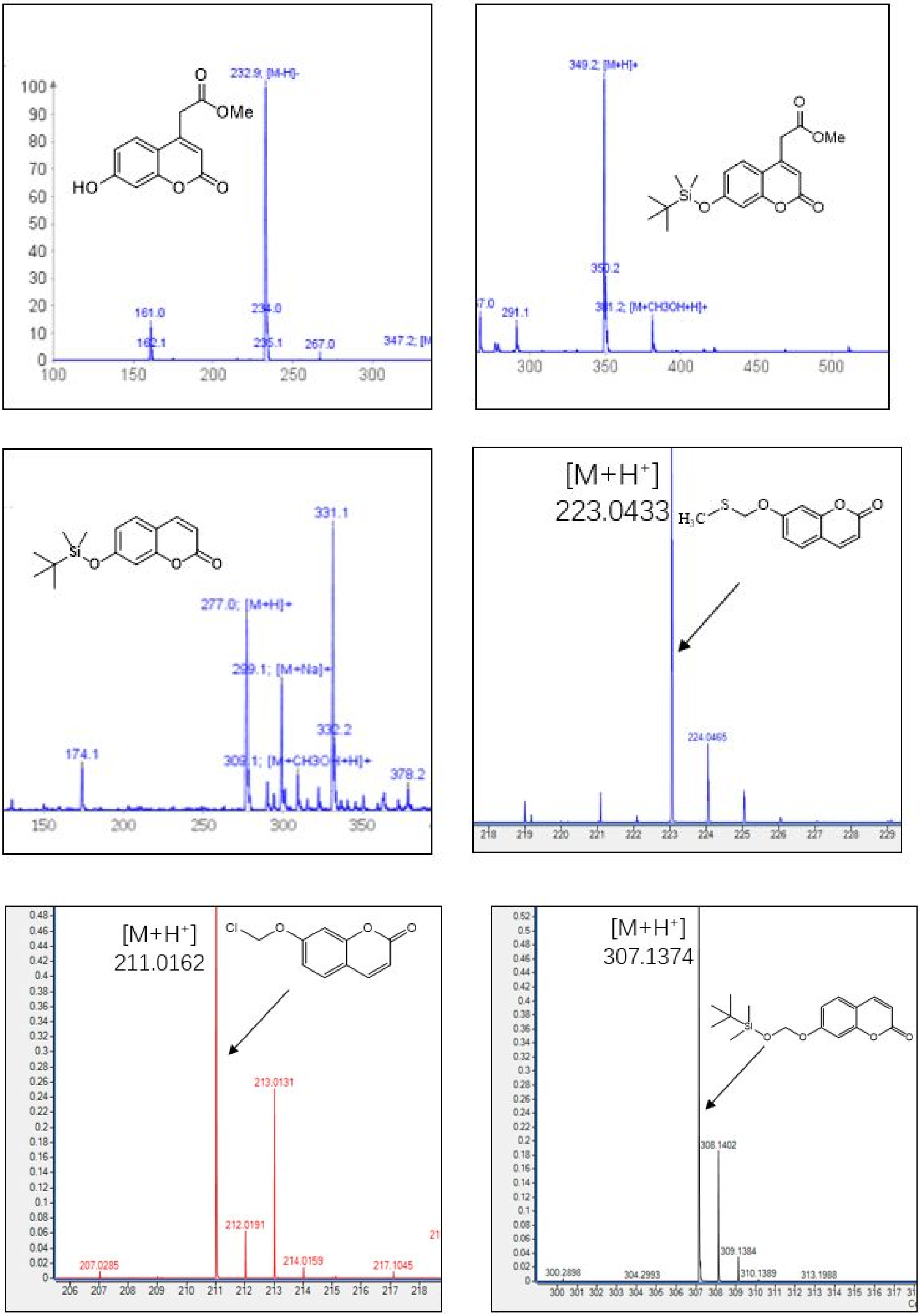

Figure S16. Mass Spectra of synthesized substrates
